# Structural and transduction patterns of human-specific polymorphic SVA insertions

**DOI:** 10.1186/s13100-025-00373-w

**Published:** 2025-11-06

**Authors:** Ashley E. Kirby, Mark Loftus, Emily C. Golba, Human Genome Structural Variation Consortium, Haley J Abel, Hufsah Ashraf, Peter A Audano, Anna O Basile, Marc Jan Bonder, Harrison Brand, Marta Byrska-Bishop, Mark JP Chaisson, Junjie Chen, Yu Chen, Zechen Chong, Nelson T Chuang, Wayne E Clarke, André Corvelo, Scott E Devine, Peter Ebert, Jana Ebler, Uday S Evani, Susan Fairley, Paul Flicek, Mark B Gerstein, Maryam Ghareghani, Ira M Hall, William T Harvey, Patrick Hasenfeld, Alex R Hastie, Wolfram Höps, PingHsun Hsieh, Sushant Kumar, Joyce Lee, Alexandra P Lewis, Chong Li, Yang I Li, Jiadong Lin, Tsung-Yu Lu, Rebecca Serra Mari, Ryan E Mills, Zepeng Mu, Katherine M Munson, David Porubsky, Benjamin Raeder, Tobias Rausch, Allison A Regier, Jingwen Ren, Bernardo Rodriguez-Martin, Ashley D Sanders, Martin Santamarina, Xinghua Shi, Oliver Stegle, Arvis Sulovari, Michael E Talkowski, Luke J Tallon, Jose MC Tubio, Aaron M Wenger, Xiaofei Yang, Kai Ye, Feyza Yilmaz, Xuefang Zhao, Weichen Zhou, Qihui Zhu, Michael C Zody, Jan O Korbel, Tobias Marschall, Evan E Eichler, Charles Lee, Miriam K. Konkel

**Affiliations:** 1 Department of Genetics and Biochemistry, College of Science, Clemson University, Clemson, South Carolina USA; 2 Clemson Center for Human Genetics, Clemson University, Greenwood, South Carolina USA

**Keywords:** Retrotransposon, Transposable element, Mobile element insertion, SVA, Subfamily, Polymorphism, Transduction, Source element, Genetic variation, Structural variation, Human specific

## Abstract

**Background:**

SINE variable number tandem repeat *Alu* elements (SVAs) are a unique group of hominid-specific composite retrotransposons with highly variable internal structure. They represent the youngest TE family in humans and contribute to genetic diversity, evolution, and disease. Recent findings indicate that SVA mobilization rates may exceed previous estimates, and many SVAs exhibit insertion polymorphism. SVAs facilitate transduction (TD) events when transcription initiates upstream of a source element, or when their internal termination signal is bypassed, mobilizing adjacent 5’ and/or 3’ sequence. To investigate features of non-reference SVA elements currently polymorphic in the human genome, we analyzed a structural variant callset built upon 35 diverse human genomes generated by the Human Genome Structural Variation Consortium.

**Results:**

In our curated dataset of 543 polymorphic, non-reference SVAs, we identify insertions representing the three youngest subfamilies: D (7%), E (38%), and F (55%). Of the latter, we determine that at least 47% are actually SVA_F_1_, a more recently discovered human-specific subfamily, indicating that F_1_ is a major contributor to SVA expansion in the human population. We further uncover that 40% of non-reference SVAs carry a TD on their 5’ and/or 3’ ends. Of these, the majority (69%) harbor sequence originating in a gene, including 14 exonic events and the mobilization of a processed pseudogene, supporting the role of SVA in exon shuffling. In addition, we identified a so-called “orphan” TD, defined by the absence of SVA sequence at the insertion site. Leveraging TD origin coordinates, we identify 55 active source elements, including nine non-reference and 46 across GRCh38 and T2T-CHM13, giving rise to 84% of TD-carrying SVAs.

**Conclusions:**

Our analyses indicate that SVA_F_1_ is more active than previously described and is a main driver of SVA expansion. We find two-fold more TD events compared to previous estimates, with an unexpected bias toward 3’ events. Finally, we postulate that the discrepant SVA mobilization rate may be attributed to inter-individual variation in the presence/absence of source elements, a recent uptick in mobilization supported by overall low allele frequencies, and/or negative selection against deleterious insertions.

**Supplementary Information:**

The online version contains supplementary material available at 10.1186/s13100-025-00373-w.

## Background

Transposable elements (TEs), mobile units of DNA, are ubiquitous across almost all eukaryotic genomes [1] and are estimated to comprise up to two thirds of the human genome [2–4]. In humans, only three TE families are currently propagating, all of which are non-LTR (long terminal repeat) retrotransposons that mobilize via an RNA intermediate [5–7]. The autonomous L1 (long interspersed element 1) provides the enzymatic machinery for its own integration, as well as for the insertion of the nonautonomous *Alu* and SVA (SINE-variable number tandem repeat-*Alu*) elements [8–10]. Of these, SVA is the evolutionarily youngest TE family, forming in the common ancestor of apes and undergoing expansion in the great apes [7, 11, 12].

SVA elements are unique due to their composite structure, with full-length insertions comprising five distinct units derived from repetitive sequences. Full-length elements are typically 1.5-3 kb in length, though their size is highly variable due primarily to copy number variation in the VNTR and hexameric regions [12]. No internal SVA promoter has been identified, but excision of the hexameric and *Alu* regions in SVA reporter constructs severely attenuates retrotransposition efficiency, suggesting promoter activity in the 5’ region [10, 13]. Given the presence of associated features, SVAs are thought to be transcribed by RNA polymerase II and inserted into the genome via L1-mediated target-primed reverse transcription (TPRT) [8–14]. SVA elements exhibit an overall GC composition of ~ 60%, exceeding 70% in the VNTR [12, 15]. Due to their GC-rich nature, SVAs have been associated with alterations in local chromatin structure and gene expression [16, 17], as well as with the predicted formation of G-quadruplex secondary structures, which have been shown to impact transcription and genome stability [18, 19]. Furthermore, SVAs are associated with altered splice patterns, which may result in differential gene expression [20–22]. Taken together, SVA elements demonstrate immense potential to shape genome structure and function.

In the lineage leading to human, SVAs are commonly divided into six subfamilies (SVA_A – SVA_F), with A representing the oldest and F the youngest subfamily [12]. Three parallel studies identified an additional, human-specific subfamily known as SVA_F_1_, comprised of at least 84 reference (NCBI35/hg17, NCBI36/hg18, and GRCh37/hg19) SVA elements [23–25]. These insertions, characterized by replacement of the hexameric, and the majority of the *Alu*, region with *MAST2* (microtubule associated serine/threonine kinase 2) exon 1 sequence, are likely the result of a non-recurrent ancestral splicing event. [23–25]. Based on whole genome analyses, SVAs are generally thought to be the retrotransposon family with the lowest mobilization rate in the human population, with about 1 new insertion per 916 live births [26]. However, a far higher rate of 1 insertion per 63 births was more recently suggested, based on pedigree analyses [27].

A subset of SVA elements co-mobilize host sequence on their 5’ and/or 3’ ends, commonly referred to as transductions (TDs), a feature first described for L1 elements [11, 23–25, 28–33]. While 5’ TDs are attributed to upstream promoters and transcription start sites (TSS), 3’ TDs result when the internal polyadenylation signal is bypassed in favor of a downstream signal [11, 23–25, 28–31]. Although the overall TD rate estimated for SVA (14–18%) is on par with that described for L1 (10–23%), their 5’ and 3’ rates differ, with the vast majority of L1-associated TDs being 3’ events and SVAs more commonly thought to harbor 5’ TDs [11, 23–25, 28–35].

Here, we utilize a non-reference SVA callset built upon 35 individuals to conduct a comprehensive analysis of structural features and elucidate subfamily propagation dynamics within humans. Further, we investigate the prevalence of SVA-mediated TD events and examine the genomic context of transduced sequences. Finally, we utilize TD sequences to identify active source elements driving SVA amplification and examine inter-individual variation in source element abundance.

## Results

### Distribution of non-reference SVA insertions

We first identified 583 putative SVA insertions from the Freeze 4 structural variation (SV) callset, generated by the Human Genome Structural Variation Consortium (HGSVC, phase 2) (Table S1), using L1ME-AID (L1-Mediated Annotation and Insertion Detector) (see *Methods*) [36]. For all putative insertions, we retrieved 500 bp of up- and downstream flanking sequence from the assemblies to identify endonuclease cleavage sites and TSDs. We then queried up- and downstream flanking sequences against the human reference genome (GRCh38) using BLAT [37] to confirm the absence of each insertion from the reference. Applying these constraints, we curated a final dataset of 543 high-confidence, non-reference SVA insertions (Table S2).

**Fig. 1:**
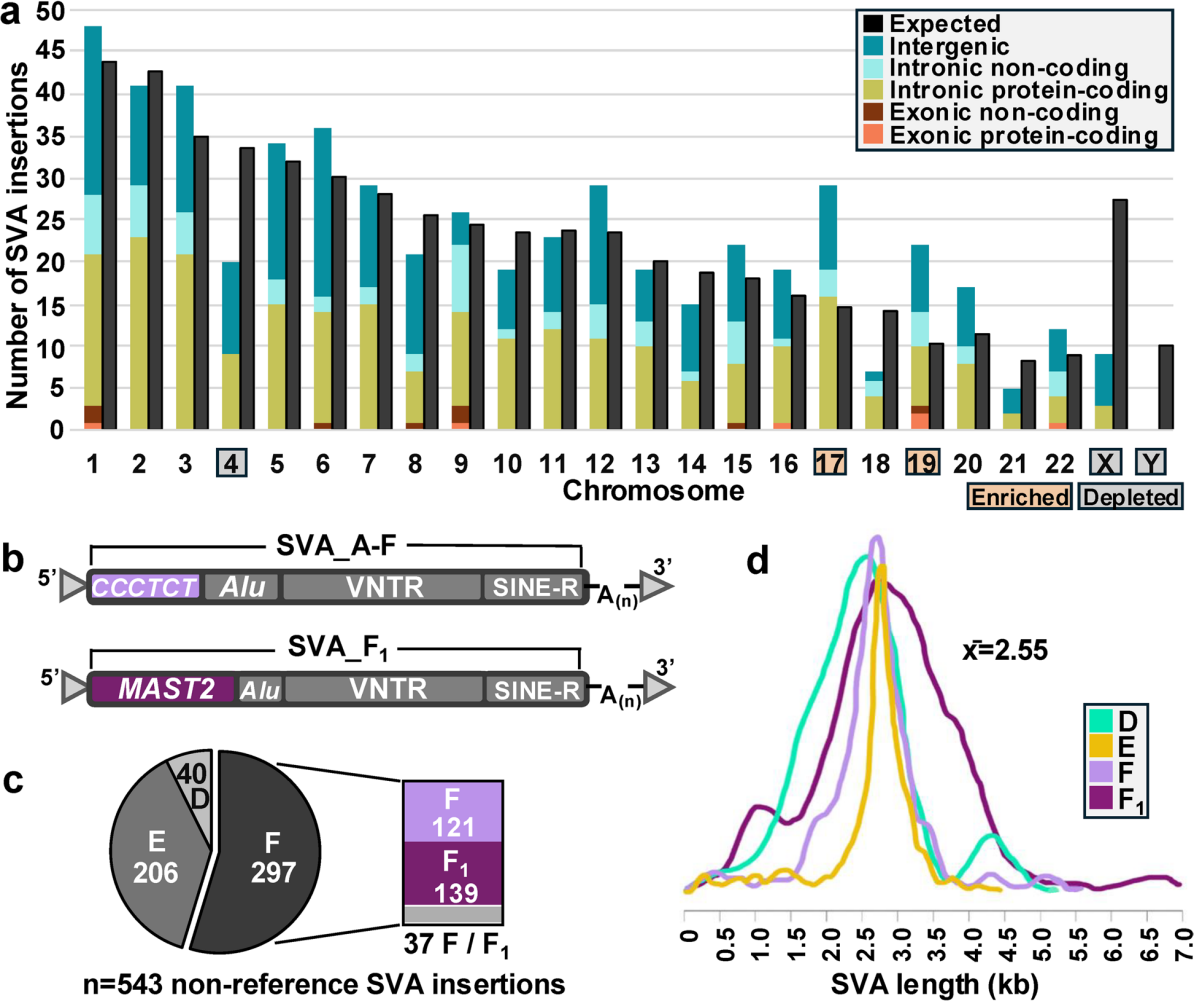
SVA element structure and insertion characteristics. **a** Genomic distribution of non-reference SVA elements. The proportion of insertions into intergenic regions, non-protein-coding genes, and protein-coding genes is shown. **b** SVA structures. Top: SVA A-F element. From 5’ to 3’, these units are commonly described as: (1) a hexameric region, composed of a variable number of CCCTCT repeat units; (2) an *Alu*-like region comprised of two antisense *Alu* fragments; (3) a variable number of tandem repeats (VNTR) region; (4) a short interspersed element of retroviral origin (SINE-R) region derived from the 3’ end of the *env* gene and the 3’ LTR of the endogenous retrovirus HERV-K10; and (5) a homopolymeric tract of adenosines (i.e., A-tail) [12]. Bottom: An SVA_F_1_, where the hexameric region, and most of the *Alu* region, is replaced with *MAST2* exon 1 sequence as the result of an ancestral splicing event from exon 1 of the *MAST2* gene to a cryptic splice site within a polymorphic SVA_F [24, 25]. **c** Subfamily annotation of non-reference SVAs. Finer-scale resolution of SVA_F revealed that the majority of insertions are SVA_F_1_. **d** Length of non-reference SVA elements by subfamily. Insertions ranged in size from 140 bp to 6.7 kb. By subfamily, SVA_F_1_ elements were the longest on average (M: 2.88 kb, Mdn: 2.88 kb), followed by SVA_F (M: 2.56 kb, Mdn: 2.63 kb), SVA_E (M: 2.55 kb, Mdn: 2.74 kb), and SVA_D (M: 2.42 kb, Mdn: 2.39 kb)

Next, we investigated the distribution of SVA insertions across chromosomes, which revealed significant deviation from the expected distribution (Chi-square test; *X*^2^ = 72.45, *df* = 23, *p* < 0.01) (Fig. 1a). In particular, we discovered a depletion of insertions on chromosomes 4, X, and Y, and an enrichment on chromosomes 17 and 19 (Fig. 1a). Leveraging the RepeatMasker annotation of GRCh38 [38], we also determined that 55% (297/543) of insertions occurred into TE sequences, with TE class breakdown in alignment with TE background composition in the genome (Table S2; Fig. S1).

Intersection of SVA insertion coordinates with GENCODE information (v46) [39] further revealed that 59% (320/543) (Table S2) of non-reference elements inserted into a gene (protein-coding: 254, 47%; non-coding: 66, 12%). Notably, insertion into protein-coding genes occurred more frequently than expected (Chi-square test; *X*^2^ = 7.49, df = 1, *p* < 0.01) based on a genomic proportion of 41% (~ 40% intronic and ~ 1% exonic) protein-coding sequence [40], further supporting a non-random distribution model. While the majority (306/320) of genic insertions were intronic, we also identified 14 exonic events (6 protein-coding and 8 non-coding), including one insertion into a 5’ UTR, 11 into 3’ UTRs (including four protein-coding genes *XBP1*, *RPSA2*, *MYCL*, and *PAGR1*), and 2 within protein-coding exons (Table S2). The latter two instances involve an insertion into *SARDH* (sarcosine dehydrogenase), as well as a previously documented insertion into *ZNF404* (zinc finger protein 404) [41]. Given both inserted into the terminal exon, nonsense mediated decay (NMD) would not be triggered, resulting in expression of proteins altered at the carboxy terminus [42, 43].

### SVA characterization

Through alignment of our 543 SVA elements against the SVA_A-F consensus sequences [44] using MUSCLE (v 3.8.31) [45], we confirmed propagation of the three youngest subfamilies (D-F), with 55% (297/543) identified as SVA_F, 38% (206/543) as SVA_E, and 7% (40/543) as SVA_D (Fig. 1b-c; Table S2). While the younger, human-specific subfamilies E and F drive SVA mobilization, we also identify SVA_D insertions, which arose prior to the radiation of the human and chimpanzee lineages [12], indicating that they continue to mobilize, albeit at a low rate.

Given that another subfamily, SVA_F_1_, was previously suggested [23–25], we investigated the abundance of SVA_F_1_ in our dataset. As SVA_F_1_ is regularly not recognized as an independent subfamily, and as the Dfam library used for annotation by RepeatMasker [38] does not include an F_1_ consensus sequence [44], we manually screened the subset initially classified as F for the presence of F_1_ insertions. Toward this, we performed an MSA against the SVA_F consensus sequence [45] and manually differentiated F from F_1_ (Fig. 1b). Our alignment revealed that F and F_1_ insertions are identical downstream from the *MAST2*/SVA splice junction at nucleotide position 385 (Fig. S2). We first identified 230 elements with sequence upstream of this breakpoint, annotating 112 SVA_F insertions and 118 SVA_F_1_ insertions. Of the remaining 67 elements, which were 5’ truncated and initiated within the shared 3’ region, we distinguished 9 SVA_F and 21 SVA_F_1_ elements by leveraging F or F_1_-defining single nucleotide variants (SNVs), indels, and 3’ TDs. Only 37 elements remained ambiguous. Thus, finer-scale resolution of the SVA_F subfamily reveals that SVA_F_1_ generated at least as many, and likely more, insertions based on discernable elements (139 F_1_ vs. 121 F) (Fig. 1c).

**Fig. 2:**
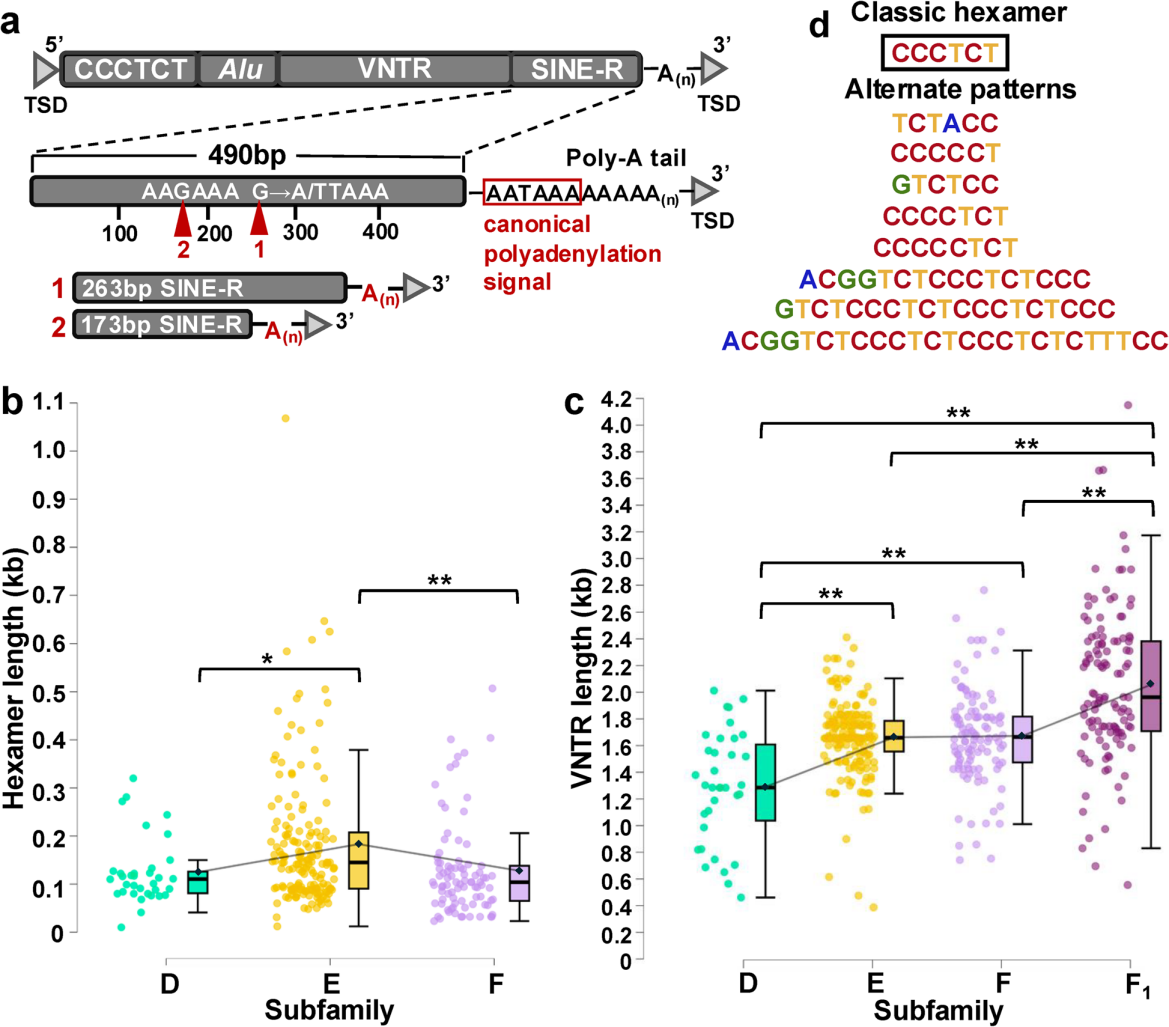
SVA structure variation. **a** Visualization of truncation hotspots identified in the SINE-R region. **b** Hexameric length distributions of full-length elements in subfamilies D (M: 125 bp, Mdn: 111 bp, *p* < 0.05), E (M: 183 bp, Mdn: 145 bp), and F (M: 126 bp, Mdn: 104 bp, *p* < 0.01). Post-hoc testing (Tukey's Honest Significant Difference (HSD)) confirmed that SVA_E hexamers were significantly longer than D (*p* < 0.05) and F *p* < 0.01) hexamers. **c** VNTR length distributions across subfamilies D-F_1_. The longest VNTRs were observed in SVA_F_1_ (M: 2057 bp, Mdn: 1963 bp), followed by F (M: 1673 bp, Mdn: 1665 bp), E (M: 1662 bp, Mdn: 1659 bp) and D (M: 1290 bp, Mdn: 1285 bp). Pairwise comparison of all subfamilies revealed that VNTR lengths vary significantly across all combinations (*p* < 0.01), with the exception of E and F. However, an increase in mean and median VNTR length from E to F was still observed. Bars denote statistically significant relationships (**p < 0.01, *p < 0.05). **d** Comparison between the classically described hexamer and alternate patterns observed in polymorphic elements of subfamilies D-F

Further analysis revealed that insertions span an average of 2.6 kb (Mdn: 2.7 kb) (Fig. 1d), and that 60% (325/543) are full length (see *Methods*; Fig. S3). As expected, alignment against SVA consensus sequences uncovered a substantially greater quantity of 5’ truncated elements (91%; 199/218) compared to those that were 3’ truncated (6%; 12/218) or truncated on both ends (3%; 7/218; confirmed to have proper A-tails) (Fig. S3). Most (90%; 17/19) 3’ truncation events clustered around two non-canonical termination sites in the SINE-R region (Fig. 2a; Table S3), which has previously been suggested to contain cryptic polyadenylation signals [12, 33].

The first cluster includes a group of eight SVA_F elements that terminate 233 bp upstream of the last nucleotide in the canonical polyadenylation signal. All but one share a substitution located 23 bp before the termination point, generating a cryptic polyadenylation signal ((G → A)TTAAA) (Fig. 2a; Table S3). A BLAT query against GRCh38 revealed six additional substitution-carrying 3’ truncated SVA_F elements in the reference genome, providing further evidence for the association of the substitution with recurrent truncation. However, the substitution may not introduce a strong polyadenylation signal, as we also identified eight SVA_F elements harboring the substitution without 3’ truncation. The second cluster of truncated SVAs terminates 323 bp upstream of the canonical signal and contains nine elements across subfamilies D, E, and F_1_ (Table S3). Truncation appears to be the result of a weak non-canonical polyadenylation signal (AAGAAA) within the SVA consensus sequence, located 17 bp upstream from the point of termination (Fig. 2a), resulting in recurrent 3’ termination across subfamilies. Only one SVA 3’ truncated in the SINE-R region terminated outside of the described hotspots.

We also investigated variation within the hexameric and VNTR regions, as well as in poly-A tails. We determined that the hexameric region ranged from 10 to 1068 bp (M: 158 bp, Mdn: 122 bp), with significant length variation across subfamilies D, E, and F (ANOVA; F(2, 284) = 7.59, *p* < 0.001), and the longest hexamers observed in SVA_E elements (Fig. 2b). VNTR lengths for all elements (D-F_1_) with a complete VNTR (i.e., not truncated within the region) ranged from 387 to 4150 bp (M: 1740 bp, Mdn: 1696 bp) and also exhibited significant variation (ANOVA; F(3,439) = 40.79, *p* < 0.001) (Fig. 2c). We noticed an inverse relationship between VNTR size and evolutionary age (i.e., younger elements harbor longer VNTRs), indicating that expansion predominates over contraction and that longer VNTRs are not prohibitive for mobilization. Finally, poly-A tails ranged from 8 to 150 bp (M: 38 bp, Mdn: 35 bp) (Fig. S3). Of note, we found that the majority (70.5%; 383/543) of A-tails contained at least one substitution, while 29.5% of A-tails were uniform.

Our investigation of the hexameric region of non-reference SVA_D-F elements revealed considerable recurrent deviation from the generally reported (CCCTCT)_n_ repeat pattern (Fig. 2d) [11, 12]. To our knowledge, only one such hexameric deviation has been previously noted: a (CCGTCT)_n_ repeat, where the “G” was attributed to reverse transcription of the 5’ cap and expansion of this addition with the hexamer [20]. Reporting only patterns shared by a minimum of five elements, we identified three hexameric variants and multiple patterns deviating from the classically described hexamer structure, including a 7-mer, 8-mer, 16-mer, 19-mer, and 25-mer (Fig. 2d). We queried GRCh38 and determined that all patterns are also present within reference SVA elements. Furthermore, we found evidence of all but one pattern (GTCTCC) in chimpanzee (panTro6), indicating that these patterns are abundant and not of recent, human-specific origin.

### SVA-mediated transductions

**Fig. 3:**
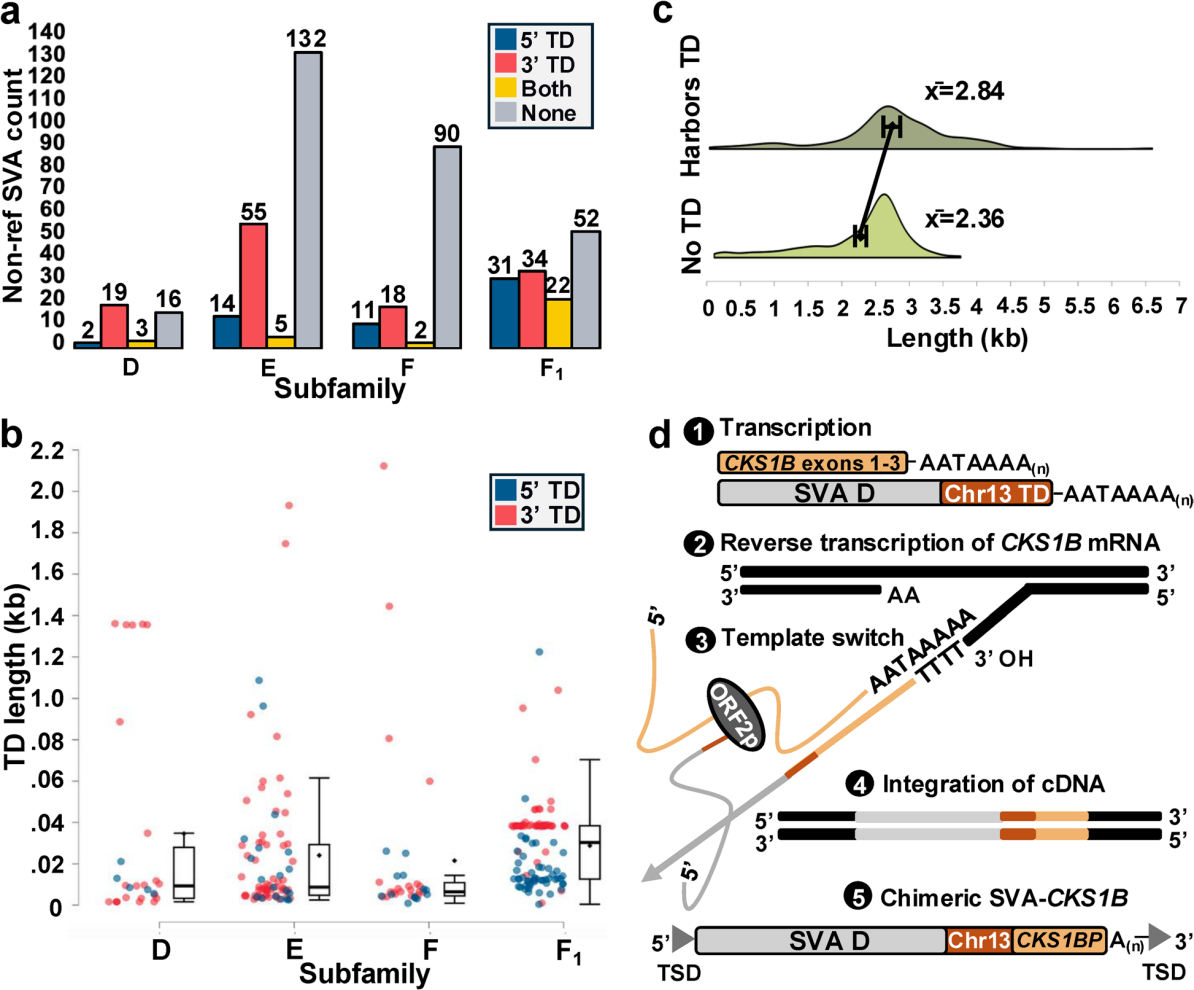
SVA-mediated TD events. **a** Number of elements carrying a 5’ TD, 3’ TD, both, or none across all subfamilies. One additional 5’ event, not shown here, is associated with a 5’ truncated SVA that could not be differentiated between subfamilies F and F_1_. **b** Distribution of 5’ and 3’ TD lengths. All TD sequences were separated into 5’ and 3’ events, with each data point representing a separate event. Investigating subfamily differences, we noted that TDs carried by SVA_F_1_ elements (Mdn: 383 bp) were 3–5 × longer than those carried by D (Mdn: 96 bp), E (Mdn: 114 bp) and F (Mdn: 74 bp) elements. **c** Comparison of non-reference SVA lengths with and without TD sequence. **d** Proposed generation mechanism of SVA-*CKS1BP*. 1) Transcription produces *CKS1B* and SVA mRNA. 2) We propose that the *CKS1B* transcript undergoes reverse transcription utilizing the L1 machinery. 3) ORF2p switches from the *CKS1B* transcript to the SVA_D transcript, and reverse transcription continues through the 5’ end of the SVA_D. 4) The chimeric cDNA product is integrated into the host DNA. 5) The chimeric SVA-*CKS1BP* is a source element, producing additional copies

We next investigated SVA-mediated TDs by aligning all non-reference SVA insertions, along with 500 bp of up- and downstream flanking sequence, and scrutinizing the 5’ and 3’ ends of each SVA for the presence of additional sequence enclosed within TSDs (see *Methods*; Table S4). While the *MAST2* sequence of SVA_F_1_ is commonly considered a TD [23–25, 32], we regard *MAST2* as a characteristic of the standard SVA_F_1_ structure, given that all SVA_F_1_ elements are likely of single origin, rather than recurrent splicing. Thus, only sequence up- and/or downstream of the F_1_ consensus sequence was considered a TD. Each putative TD was queried against GRCh38 using BLAT [37] to determine its source locus and to identify additional putative elements with TDs. We determined that 40% (217/543) of the SVA insertions in our dataset harbor a TD. Of these, 58 (27%) carry a 5’ TD and 127 (58%) harbor a 3’ TD (Fig. 3a; Table S4), indicating that 3’ events occur about 2.2 × more frequently than 5’ events. In addition, we found that 32 (15%) elements have both a 5’ and 3’ TD (Fig. 3a; Table S4).

Subfamilies exhibited variable TD rates, with 60% (24/40) of D, 36% (74/206) of E, 26% (31/121) of F, and 63% (87/139) of F_1_ elements carrying a TD (Fig. S4). While TDs were, on average, 284 bp in length, 3’ TDs were about two-fold longer (M: 347 bp, Mdn: 258 bp) than 5’ TDs (M: 172 bp, Mdn: 124 bp), a statistically significant difference (Student’s t-test; *t*(230.3) = −4.04, *p* < 0.001) (Fig. 3b). Lengths ranged from 9 bp, to as long as 4 kb. Ultra-short 3’ TDs were observed and represent mobilization events of the source element together with its TSD. These events were confirmed through alignment of non-reference insertions to their source element and flanking sequence. Overall, the average length of non-reference SVAs without a TD was 2.36 kb, compared to 2.84 kb for those with a TD (Fig. 3c). Furthermore, we observed that SVAs with a 3’ TD generally contained significantly shorter poly-A tails (M: 29.1 bp, Mdn: 26 bp) (ANOVA; F(2, 540)) = 28.48, *p* < 0.001), compared to those with a 5’ TD (M: 42.1 bp, Mdn: 42.5 bp) or no TD (M: 41.5 bp, Mdn: 39 bp) (Fig. S4).

TDs were traced to all chromosomes, except 18, 21, and Y (Table S4). We identified 27 cases in which TD sequence from multiple source loci was carried by a single SVA (Fig. S5; Table S4), referred to here as concatenated events, demonstrating that offspring elements serve as source elements and that TDs accumulate sequentially [11, 24, 32]. Interestingly, we also observed a TD-only insertion (i.e., absence of SVA), which is caused by premature termination of the reverse transcription process. Such events, deemed “orphan” TDs, have been described for L1 [46], but not for SVA.

### Transduction groups

We next screened all 217 TD-containing SVAs for the presence of shared TD sequence, assigning 68% (149/217) to 25 groups of two or more elements (Table S6). This includes six 5’ TD groups encompassing 15 insertions, and 18 3’ TD groups containing 69 insertions. In addition, we observed 65 SVA_F_1_ insertions carrying TD sequence (5’ = 15; 3’ = 28; both = 22) derived from a locus on chromosome 10, previously associated with 13 SVA_F_1_ elements in the human reference genome [23–25] (Table S5; Fig. S5). Of these, five represent concatenated events, carrying TDs from chromosomes 8 (3’), 14 (*n* = 2; 3’), 17 (both), and 19 (5’) in addition to chromosome 10 sequence (Table S5). Based on this, we can infer that chromosome 10-derived SVA_F_1_ elements have been mobilized by at least six source elements. SVA_F_1_ elements derived directly or indirectly from chromosome 10 represent, by far, the largest TD group in our dataset, and the only group including insertions with both 5’ and 3’ shared sequence (Table S5).

One notable 3’ TD group included five non-reference elements composed of a full-length SVA_D, an 87 bp L1M5 fragment, and the VNTR and SINE-R region of a 5’ truncated SVA_F (Fig S6). A BLAT [37] query of this concatenated SVA against GRCh38 traced the source locus to chromosome 14 (chr14:22,634,849). Further analysis revealed two independent insertions (confirmed by the presence of unique TSDs enclosing each element), with the SVA_D inserted upstream, and the SVA_F downstream, of the L1M5 sequence. While the latter sequence is shared with panTro6, the SVA insertions are human specific. The overall higher frequency of 3’ TDs, their propensity to be longer than 5’ TDs, and the fact that the SVA_D is full length suggests that the L1M5 and SVA_F sequences are carried by the SVA_D as a 3’ TD.

### Analysis of transduction sequence

We found that 68% (148/217) of TDs contain TE sequence based on RepeatMasker annotation (Table S7; Fig. S5) [38], including 13% (28/217) with multiple TEs. Most TE sequences represent fragments rather than full-length elements. All [47] chromosome 10-derived SVA_F_1_ elements include transduced *Alu* sequence on the 5’ and/or 3’ end, and 64% (14/22) of SVA_F_1_ TDs not associated with chromosome 10 also harbored some *Alu* sequence. Thus, 91% (79/87) of all SVA_F_1_ insertions with a TD acquired *Alu* sequence, equating to 70% of SVA_F_1_ insertions overall. Excluding chromosome 10-derived elements, we find that 68% (36/53) of 5’ TDs contain TE sequence, compared to 49% (53/109) of 3’ events. Thus, 5’ TDs appear to be slightly enriched for TE sequence, while 3’ TDs are consistent with the genome background. Furthermore, 5’ TDs show ~ 4 × enrichment for *Alu* (21 loci) and ~ 3 × for ERV (14 loci), compared to background proportions. This may suggest that acquisition of SINE and/or LTR sequences on the 5’ end is beneficial to transcription and/or propagation. Considering all TDs, TE family composition largely corresponded to the genomic background, with the exception of *Alu*, for which we found an overall enrichment approximately five-fold greater than background levels (Fig. S5).

Analysis of TD source locations revealed that the majority (69%; 150/217) originate within a gene, including 125 protein-coding and 25 non-protein-coding (Table S7), representing a significant enrichment compared to both the total genome composition (based on a proportion of 50.2% genic sequence) [48] (Chi-square test; *X*_2_ = 31.09, *df* = 1, *p* < 0.001) and the distribution of non-reference insertions in our dataset (Chi-square test; *X*_2_ = 9.195, *df* = 1, *p* < 0.01). The vast majority of TDs (140; 93%) contain intronic sequence, while ten (7%) map to exonic sequence (Table S7). Of the latter, five are from four protein-coding genes (*CASP8*, *MRPS18A*, *UQCRC1*, and *HGSNAT*), three originate from two antisense long non-coding RNA genes (*TSPAN14-AS1* and *DCTN1*-*AS1*), and the remaining two map to a long intergenic RNA gene (*LINC02916*) and a close to full-length processed pseudogene (*CKS1BP*) (Fig. 3d). With the exception of *CASP8* and *HGSNAT* [32, 49] (Fig. S7), all other exonic TDs have not been reported.

The three exonic 5’ TDs (*LINC02916*, *MRPS18A*, *UQCRC1*) are generated by splicing, either from an exon into an SVA (*MRPS18A*) (Fig. S8), or canonical splicing between exons 1 and 2, followed by readthrough into intron 2 and termination at the 3’ end of the SVA (*LINC02916* and *UQCRC1*) (Fig. S9). The *MRPS18A*-containing TD initiates 5 bp upstream of exon 1 and transitions from the end of exon 1 into the SVA at the same splice site within the *Alu* region that gave rise to SVA_F_1_ [25], supporting splicing at this position as a recurrent mechanism (Fig. S8). While most 3’ TDs (*HGSNAT*, *CASP8* (*n* = 2), *TSPAN14*-*AS1* (*n* = 2), *DCTN1*-*AS1*) are the result of polyadenylation signal readthrough, one insertion (*CKS1BP*) appears to be generated by a different mechanism.

A BLAT [37] query of an 897 bp 3’ TD against GRCh38, revealed that 121 bp immediately following the SVA mapped to chromosomes 11 (73,935,481) and 13 (98,030,050), while the 3’ end of the TD mapped to eight loci. Of these, seven represent processed pseudogenes, and the remaining locus constitutes the exons of the *CKS1B* gene, beginning at the terminal half of exon 1. First investigating chromosomes 11 and 13, we used RepeatMasker to search for a reference SVA within 5 kb of the 121 bp TD sequence [38]. In doing so, we identified an SVA_D upstream from the TD sequence on chromosome 11, with 14 bp TSDs enclosing both the SVA and the TD. Based on this, and absence of both at the panTro6 orthologous site, we ruled out chromosome 11 as the origin locus. In contrast, we identified the 121 bp TD sequence on chromosome 13 in both human and chimpanzee, indicating that it is the origin locus. Based on these findings, we infer that an SVA_D inserted on chromosome 13 but was either lost from the human population or is present at a low allele frequency.

To further investigate the origin of the *CKS1BP* sequence within the 3’ TD, we performed an MSA of each *CKS1BP* hit in GRCh38, plus 5 kb bp of up- and downstream flanking sequence [45]. This revealed that two copies, *CKS1BP2* (chr10:29,697,935) and *CKS1BP3* (chr5:62,511,771), harbor SVA_D elements upstream of the *CKS1BP* sequence and, hence, share the same TD as the non-reference SVA. Mobilization as a unit was further substantiated by the presence of unique TSDs encompassing each SVA and its TD. The five additional processed pseudogenes were neither associated with SVA sequence nor the TD sequence derived from chromosome 11.

To elucidate the origin of the *CKS1BP*-containing SVA elements, we generated a maximum likelihood tree [50, 51] based only on shared sequence across all *CKS1BP* copies and the *CKS1B* mRNA (Fig. S11). Our tree revealed that the non-reference SVA shares the greatest homology with *CKS1BP3*, followed by *CKS1BP2*; and that SVA-associated and non-SVA-associated *CKS1BP* copies form two distinct clades (Fig. S11). Substantiating these relationships, a BLAT query of *CKS1BP* flanking sequences against panTro6, bonobo (panPan3) and gorilla (gorGor6) [37], revealed that only the five non-SVA-associated copies are present at orthologous loci. Thus, the three SVA-associated copies appear to be of more recent, human-specific origin.

Given the absence of a poly-A tail separating the concatenated 3’ TD events, the lack of an identifiable splice site (confirmed via manual inspection and a predictive tool [52]), and 5’ truncation of the *CKS1B* sequence within the TD, we propose that a template switch from the *CKS1B* mRNA to the SVA mRNA during reverse transcription generated the first SVA-*CKS1BP* copy (Fig. 3d). Based on phylogenetic analysis and sequence scrutiny, we speculate that *CKS1BP2* is the source of our non-reference SVA, which subsequently produced *CKS1BP3*. Support for this includes a short, concatenated 3’ TD carried by the non-reference SVA, traced to *CKS1BP2*, as well as unique substitutions shared only between the non-reference insertion and *CKS1BP3*. Our findings demonstrate that SVA-mediated TD represents an alternate pathway for pseudogene propagation.

### Identification of source elements

**Fig. 4:**
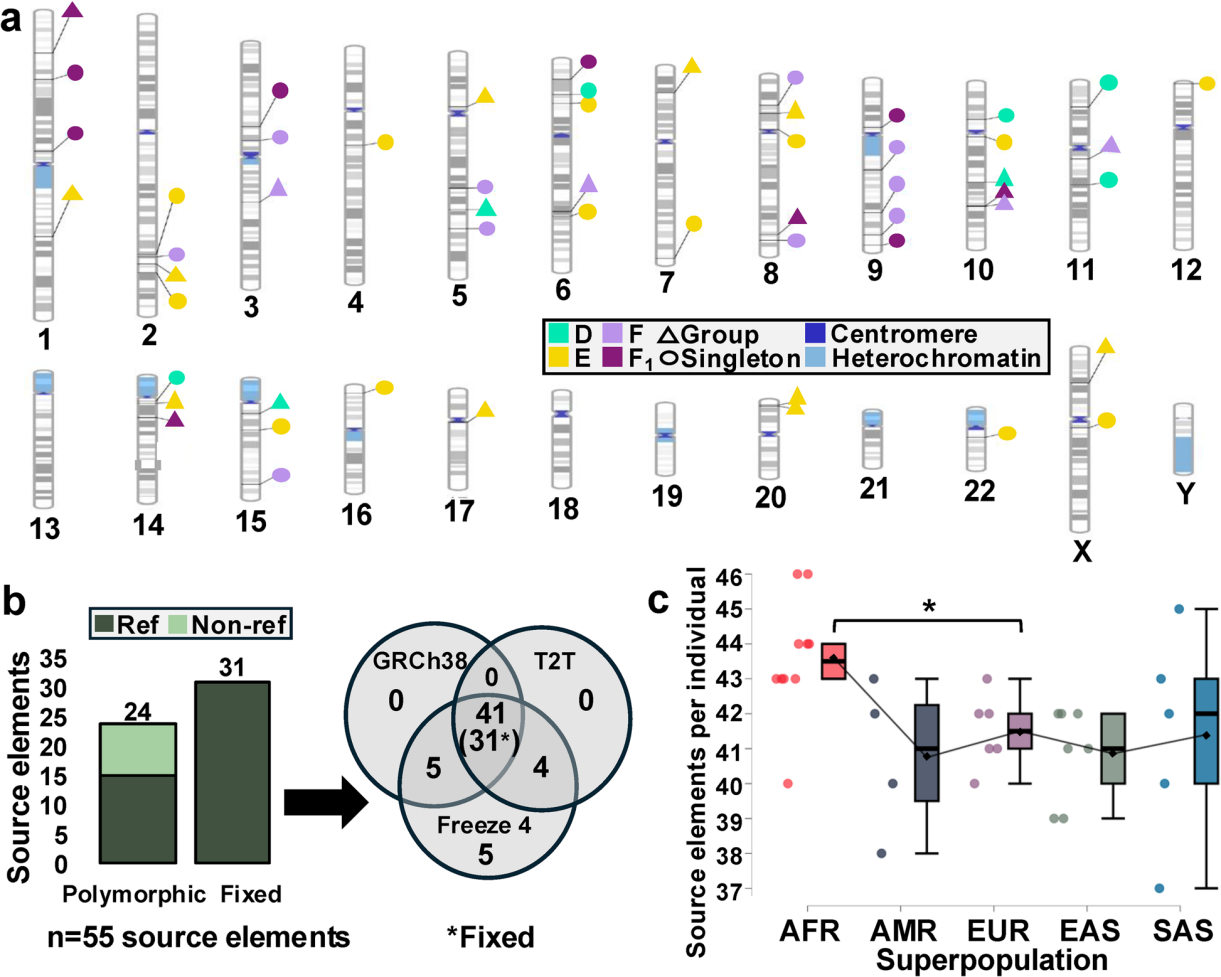
SVA TD groups and source elements. **a** Chromosome ideogram depicting loci at which source elements for 21 TD groups (triangles) and 34 singleton TD events (circles) were identified. No source elements were identified on chromosomes 13, 18, 19, 21, or Y. However, the identification of six SVA elements with TDs from chromosome 19 and two from chromosome 13 indicate that there were polymorphic source elements on these chromosomes at some point. **b** Polymorphic and fixed source elements were identified across GRCh38, T2T-CHM13, and the Freeze 4 callset. Reference vs. non-reference refers to presence in GRCh38. **c** Number of source element insertions per individual, including fixed and polymorphic source elements across GRCh38, T2T-CHM13, and Freeze 4. An ANOVA revealed significant variation (F(4, 27)) = 3.137, *p* < 0.05) between groups, though AFR and EUR were the only significant pairwise comparison (*p < 0.05; Tukey’s (HSD))

Leveraging TD origin coordinates, we searched our non-reference dataset, GRCh38, and T2T-CHM13 for source elements up- or downstream of the TD origin. We identified 9 non-reference source elements in our dataset giving rise to 14 insertions, and 46 additional source elements across GRCh38 and T2T-CHM13 (Fig. 4a-b; Table S8). These 55 source elements gave rise to 183 insertions in our dataset, including 21/25 TD groups and 34/67 singleton events, representing 84% of TD-containing insertions (Table S8). For the remaining insertions, we can infer the origin of the TD despite the absence of an SVA, which may be lost from the population or present at low allele frequency.

We then investigated inter-individual differences in source element abundance across 32 individuals (3 offspring from trios were excluded). Intersecting the insertion coordinates of the 55 identified source elements with the Freeze 4 DEL callset, which encompasses loci that are absent in at least one individual relative to GRCh38, we identified 15 reference source elements that are polymorphic in our individuals. In combination with the nine non-reference source elements identified within our SVA dataset, we uncovered a total of 24 polymorphic source elements, with allele frequencies ranging from 0.016 to 0.95 (M: 0.32, Mdn: 0.38). The remaining 31 reference insertions were homozygous present in all individuals, indicating that they are (nearly) fixed in the human population (Fig. 4b; Table S8).

Of the 55 source SVAs, almost all [53] were present within the AFR population, with the next greatest quantity in the AMR and SAS populations [49], followed by EUR and EAS [48]. Individuals harbored between 37 and 46 source elements, with the highest count observed in two individuals of African ancestry (HG03065 and NA19239) and the lowest in an individual of South Asian ancestry (HG03683) (Fig. 4c). Thus, the greatest source element abundance was observed in the AFR superpopulation, on both a population and individual level. Investigating source element subfamily dynamics, we found that 42% were SVA_E, followed by 25% SVA_F, 15% SVA_D, and 18% SVA_F_1_ (Fig. 4a). The relatively low abundance of SVA_F_1_ source elements, despite their overall high TD rate and a propagation rate potentially exceeding SVA_F, may reflect bias stemming from the grouping of chromosome 10-derived elements. Supporting this, shared sequence features within groups of SVA_F_1_ elements, and concatenated TDs carried by chromosome 10-derived insertions, point to additional unrecovered source elements.

### SVA propagation rate and allele frequency

**Fig. 5:**
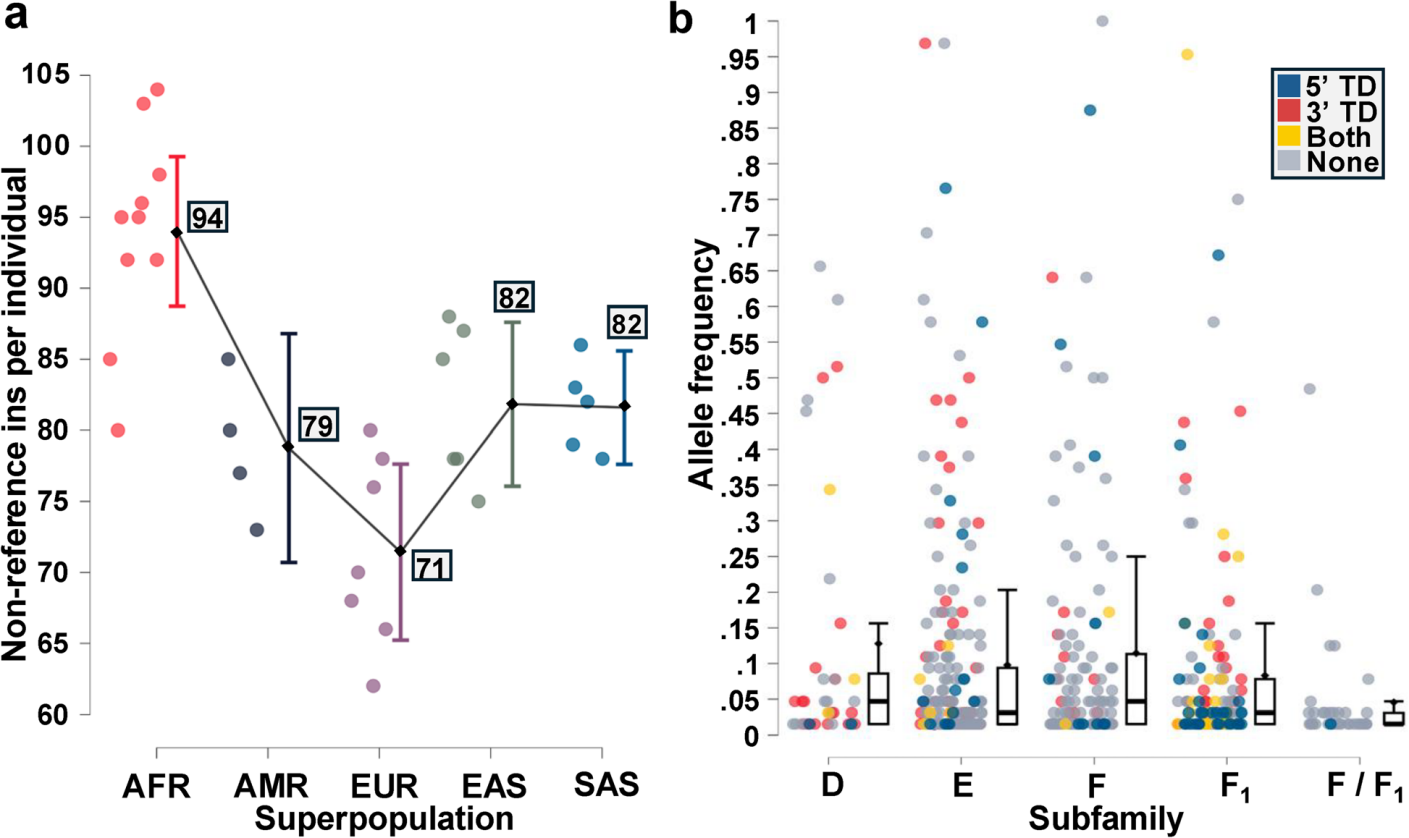
Non-reference SVA insertion counts and allele frequencies. **a** SVA propagation differences across 32 individuals (excluding three offspring within trios). The number of non-reference SVA insertions ranged from 62 to 104 (M: 83) per individual. An ANOVA test revealed significant differences in insertion count across superpopulations (F(4, 559) = 14.71, *p* < 0.001), specifically between AFR and all other groups. **b** Allele frequencies for 540 polymorphic insertions across 32 individuals, by subfamily and TD type. Insertions with missing genotypes were excluded. No significant difference in allele frequencies was observed across subfamilies D-F_1_ (median D: 0.0468, median E: 0.0313, median F: 0.0468, median F_1_: 0.0313, median F/F_1_: 0.015625). The F/F_1_ category refers to the 37 elements that could not be distinguished between F and F_1_

Next, we investigated the allele frequencies of 540 non-reference SVA insertions (complete genotype information was unavailable for 3/543) across 32 individuals (offspring from trios were excluded). We observed a mean of 83 non-reference SVA insertions per individual, with the highest and lowest abundance in AFR (M: 94) and EUR (M: 71), respectively (Fig. 5a). Two-thirds (67%; 361/540) of non-reference SVAs had an allele frequency of < 0.05 (M:0.031, Mdn: 0.096) (Fig. 5b), with 47% (253/540) representing singleton events.

## Discussion

Investigating the genomic distribution of the 543 polymorphic, non-reference SVA elements, we find evidence for non-random insertion, in agreement with previous analyses [12, 16, 18]. In particular, we observe enrichment of insertions into genic sequence, which is in alignment with other studies investigating polymorphic insertions [16, 18]. However, this may be attributed more to the accessibility of open chromatin during SVA mobilization than to targeted insertion into genic regions [18]. In contrast, for reference genome insertions, no such bias was observed [12], indicating that genic insertions may be lost due to negative selection. While we did not find a distribution bias based on allele frequency overall, as expected, the majority (12/14; 86%) of exonic insertions were present at very low allele frequencies (9/14: 1.6%, 3/14: 7.8%). Most (11/14; 79%) exonic SVAs were located in the 3’ UTR, in line with findings that insertions occur more frequently than expected in this region [22, 53, 54]. Such insertions have been associated with reduced expression of protein-coding genes [21, 53].

Also in alignment with previous observations [12], we found that 60% of SVAs are full length and that VNTR length is inversely correlated with evolutionary age. Expansion of the VNTR over time may be the result of polymerase slippage during replication or transcription, or ORF2p slippage during reverse transcription [20], supporting a pattern of expansion rather than contraction. Our fine scale-analyses revealed considerable deviation from the canonical hexameric pattern, with most of the observed patterns likely being introduced prior to the radiation of the human and chimpanzee lineages. Given the hexameric region is suspected to possess promoter activity and has been identified as a source of G-quadruplex formation [13, 18, 55], functional studies exploring whether the observed patterns influence mobilization may provide additional insight into the role of the hexameric region in SVA transcription and retrotransposition.

Our annotation of non-reference SVA insertions across 35 individuals (32 plus 3 offspring) reveals a much higher mobilization rate of SVA_F_1_ in the human population than previously appreciated. SVA_F_1_ represents only a minor fraction of SVA insertions in the human reference genome, with 84 SVAs identified as SVA_F_1_ (~ 32% of SVA_F elements) [24, 25]. In contrast, we identified 139 non-reference SVA_F_1_ elements across 32 genetically diverse humans, making SVA_F_1_ more abundant than SVA_F, based on discernable elements classically annotated as SVA_F. The enrichment of SVA_F_1_ among young, polymorphic SVA elements compared to the human reference genome suggests that SVA_F_1_ is a highly active subfamily, which is also supported by its overall low allele frequency (M: 8%). Thus, SVA_F_1_ is possibly gaining traction in the human population and may be in the process of outperforming SVA_F. Taken together, we argue that SVA_F_1_ should be considered its own subfamily and included in the standard SVA annotation. Further, given the likely origin of SVA_F_1_ via a single splicing event rather than recurrent splicing, we argue that the *MAST2* sequence should no longer be considered a TD, but part of the standard SVA_F_1_ structure.

Despite not considering the *MAST2* sequence a TD, we identify more than twice as many TDs compared to previous studies (40% vs. 14–18% with TD) [23–25, 31, 32]. Thus, our SVA_F_1_ annotation shifts the previously reported predominance of 5’ TDs to 3’ TDs being more prevalently associated with SVA elements, making it more aligned with L1 insertions, which harbor primarily 3’ TDs [28–30]. Further contributing to this is our identification of short 3’ TDs that do not appear to result from the use of downstream polyadenylation signals and often represent retention of the parent TSD in the offspring insertion. Though we speculate that these events are attributed to initiation of the poly-A tail downstream from the canonical polyadenylation signal, further investigation into the mechanism is warranted.

While L1-mediated 5’ TD events are rare [28], SVA-mediated 5’ events are more routinely observed, which may be attributed to differences in promoter usage, as L1 utilizes a well-documented internal promoter [56], yet no such internal promoter has been identified within SVA elements. Rather, both internal and external promoters likely play a role in SVA transcription [10, 13], which may explain their increased acquisition of 5’ sequences relative to L1 elements. Interestingly, we find that 91% of 5’ TDs in our dataset contain genic sequence and/or SINE and LTR elements, significantly more than would be expected based on the overall genomic distribution of SVA. Thus, we postulate that SVA insertions within these regions may be more active than insertions within other genomic environments, given closer proximity to external promoters, regulatory features, and the transcription machinery are likely beneficial to amplification.

The considerable length variation of the composite SVA structure, which commonly leads to fragmentation of SVAs, combined with the presence of 5’ and/or 3’ TDs, which can complicate SVA discovery, are likely contributing factors of SVA underascertainment. Thus, implementation of SVA_F_1_ annotation into standard repeat analyses and accurate annotation of TDs may likely improve SVA insertion discovery. For example, determination of TSDs combined with a BLAT query of any non-SVA sequence within TSDs, led to the discovery of very short TDs, which often represent the TSDs of the source element, as well as very long, concatenated sequences.

Our analyses further support acquisition of TD sequence through initiation of transcription at upstream start sites, readthrough of the 3’ end, and alternative splicing into the SVA [23–25]. In addition, our analyses reveal template switch between a processed pseudogene and an SVA, as well as canonical splicing of a long non-coding RNA, followed by readthrough into the SVA, as additional mechanisms for SVA-mediated TD. Our findings of exonic TDs and the mobilization of a processed pseudogene further elucidate the role of SVA in genome-shaping events, such as exon shuffling and gene duplication [25, 31].

Previously, the 3’ *Alu* TD associated with many chromosome 10-derived SVA_F_1_ elements was shown to increase its mobilization by 25-fold, compared to chromosome 10-derived SVA_F_1_ elements only carrying the 5’ *Alu* TD [13]. Here, we demonstrate that the majority (70%) of all SVA_F_1_ insertions, including those not associated with chromosome 10, have acquired some *Alu* sequence via a TD, supporting that inclusion may be advantageous for retrotransposition. The overrepresentation could be a consequence of the ability of *Alu* elements to capture L1 proteins at the ribosome, subsequently promoting retrotransposition [8, 57, 58] and a compensatory mechanism in response to replacement of the majority of the internal *Alu* region by *MAST2* [24]. In alignment with this, we found that only 18% of SVA_D-F elements carrying a TD had acquired *Alu* sequence (representing ~ 6% of all D-F elements in our dataset).

We also observed SVA-mediated mobilization of exonic sequence, and even of a close to full-length *CKS1B* processed pseudogene. Based on the divergence from the *CKS1B* gene combined with presence in chimpanzee, we can infer that the five non-SVA associated *CKS1BP* reference copies represent separate insertion events of processed pseudogenes mediated by the enzymatic machinery of L1. In contrast, the pattern of the three human-specific SVA-associated processed pseudogenes suggests a single origin event, followed by mobilization as an SVA-TD-processed pseudogene unit. Interestingly, *CKS1BP7*, a non-SVA-associated copy, is expressed in about a third of breast cancer tumors [59], and overexpression of the *CKS1B* parent gene is associated with aggressive progression and poor prognosis in a number of cancers [47, 60–66].

We also identified two SVA elements carrying a 3’ TD of *CASP8* exonic sequence in antisense orientation. One of these, along with an additional polymorphic insertion carrying the *CASP8* TD, had been previously identified in the Icelandic population. The source SVA element, which is present in GRCh38 and located in intron 8 of the *CASP8* gene, has been associated with aberrant splicing and intron retention [49]. Furthermore, this insertion has been associated with breast cancer and basal cell carcinoma occurrence, as well as protection against prostate cancer [49]. Finally, we identified an orphan TD, wherein the TD sequence was identified without an adjacent SVA, likely as a result of extreme 5’ truncation during reverse transcription. A pathogenic L1-mediated orphan TD was previously identified as the causative variant in Duchenne muscular dystrophy [46], demonstrating that such insertions have mutagenic potential.

We identified active, polymorphic source elements for all propagating subfamilies (D-F_1_), supporting recent activity, or even continuing propagation, in the human population and shedding light on elements with the highest potential for generating de novo insertions. We found that individuals possess between 37–46 source elements, likely an underestimation given our analysis was based solely on source elements producing TDs. Such differences in source element abundance lead to inter-individual variation in SVA propagation rates, contributing to genetic variation, evidenced by our finding that individuals possess between 62–104 total non-reference insertions. Ongoing mobilization of subfamilies D-F_1_ is further supported by the identification of at least 28 insertions causing monogenic disorders [21], and the impact of SVA on phenotypic variation, such as skin pigmentation loss in European populations [67].

Interestingly, SVA_E comprised the greatest proportion of identified source elements (42%), and of all non-reference insertions (38%; 206/543), despite comprising only ~ 4.4% of reference SVAs [12], suggesting that SVA_E is actively propagating, with potentially the highest propagation rate. The low abundance in the human reference genome may indicate a recent increase in propagation. Alternatively, SVA_E may underlie strong negative selection. In contrast, SVA_D comprised 15% of source elements and 7% (40/543) of non-reference insertions, despite representing ~ 43% of reference SVAs [12], suggesting that this evolutionarily older subfamily is currently mobilizing at far lower rates than in the past.

We identified ten SVA_F_1_ source elements. Of these, two, including the chromosome 10 master source and a non-reference, polymorphic source on chromosome 14, produced offspring with chromosome 10-derived TDs. Thus, we provide evidence that offspring produced by the chromosome 10 master locus are also retrotransposition competent. SNVs shared among small groups of chromosome 10-derived elements, and SVA_F_1_ elements carrying concatenated TDs, point to additional, unrecovered source elements that have likely been lost from the population. Given this, we speculate that the F_1_ element on chromosome 10 may be a “stealth driver,” giving rise to short-lived, but highly active, insertions that are quickly lost from the population, as previously described for *Alu* [12, 68]. The comparable number of identified F and F_1_ source elements (14 and 10, respectively), in conjunction with the potential for unrecovered F_1_ source elements, provides support for our hypothesis that F_1_ is currently propagating at levels on par with, and potentially exceeding, SVA_F.

## Conclusion

Here, we provide insight into the youngest, polymorphic SVA elements currently propagating in the human population. Our analyses reveal that the evolutionary youngest subfamily, SVA_F_1_, is establishing itself as a main driver of SVA expansion in the human population. Based on this abundance, we strongly argue that SVA_F_1_ has reached subfamily status. Further, we discovered that the overall SVA-mediated TD rate is two-fold higher than previously estimated, and that 3’ events are more abundant than 5’ events in our dataset. We highlight the wide range of mechanisms contributing to TD events beyond upstream promoter usage (leading to 5’ TD) and 3’ polyadenylation signal readthrough, such as template switch. Leveraging TD sequences, we trace 84% of TD-containing elements to 55 active source elements and highlight variation in source element abundance on an individual and population level.

## Methods

### SVA identification

A local installation of RepeatMasker (v4.1.6) [38] with the Dfam (v3.8) [44] library was run on the HGSVC Freeze 4 merged SV callset. The RepeatMasker.out file and a Fasta file containing all SV sequences were provided to L1ME-AID (v1.0.0-beta) [36] (https://github.com/Markloftus/L1ME-AID), a pipeline which identifies TEs based on presence of non-LTR retrotransposon sequence (*Alu*, L1, SVA), a poly-A tail, and semi-low divergence (*Alu*: < 6%, L1/SVA: < 15%) compared to the Dfam consensus sequence [44]. SVAs were then filtered out, producing 583 putative elements. To manually curate, 500 bp of flanking sequence up- and downstream of each insertion coordinate was pulled from the assemblies and used to confirm endonuclease cleavage sites, TSDs and poly-A tails. Flanking sequences were also queried against GRCh38 to confirm the absence of each SVA from the reference genome [37]. Ultimately, 40 putative loci were removed in accordance with our established parameters, resulting in a final high-confidence dataset of 543 non-reference SVAs.

### Sequence analyses

All sequence alignments were generated using MUSCLE (v3.8.31) [45] and visualized in AliView (v1.27) (https://github.com/AliView/AliView) [69].

### Statistical analyses

JASP software (v0.19.3) (https://github.com/jasp-stats) [70] was used to perform statistical analyses, including: Chi-square Goodness-of-Fit test, ANOVA with Tukey's Honest Significant Difference post-hoc test, and Independent Samples T-test. Figures 2b-c, 3b, 4c, and 5a-b were generated using JASP [70].

### Phylogenetic tree generation

MSAs were generated using MUSCLE (v3.8.31) [45] and resultant alignments were input into raxmlGUI 2.0 (v.2.0.15) [50] to generate maximum likelihood trees (https://antonellilab.github.io/raxmlGUI/). Phylogenetic trees were visualized using FigTree (v1.4.4) [51] (https://github.com/rambaut/figtree).

### Genomic distribution

A Chi-square Goodness-of-Fit test was performed to compare the observed (based on non-reference SVA insertion coordinates) and expected distribution of SVA elements. Expected counts were calculated by dividing each chromosome length by the total size of GRCh38 [71], and multiplying the resultant proportion by 543.

To determine if SVAs were inter- or intragenic, insertion coordinates for all 543 non-reference elements were intersected with GENCODE gene information (v46lift37) [39] and visualized on the UCSC Genome Browser (GRCh38). A Chi-square Goodness-of-Fit test was performed based on a genomic proportion of 41% (~ 40% intronic and ~ 1% exonic) protein-coding genes [40]. Exonic insertions were further scrutinized to determine insertion into coding sequence or UTRs (GENCODE v46lift37) [39]. The RepeatMasker (v3.0.1) [38] track of the UCSC Genome Browser (GRCh38) was used to evaluate insertion sites for presence of TEs.

### Subfamily annotation

SVAs were first grouped into subfamily bins (e.g., SVA D/E/F) based on RepeatMasker (v4.1.6; Dfam v3.8) annotation [38, 44]. Next, an MSA was generated for each subfamily bin, and SVA sequences were scrutinized for any deviation from the established (Dfam) consensus sequence. SVA_F elements containing *MAST2* exon 1 sequence on their 5’ ends (confirmed via a BLAT search against GRCh38 [37]) were filtered from the SVA_F group and used to generate an SVA_F_1_ subfamily-specific alignment.

### Structural analyses

For subfamilies D-F, full length was defined as the presence of at least one hexameric repeat on the 5’ end and a poly-A tail following a complete SINE-R region on the 3’ end. For SVA_F_1_, full length was defined as > 320 bp of *MAST2* sequence on the 5’ end, based on the distance from the *MAST2*/SVA splice junction to the beginning of the first hexameric repeat relative to the SVA_F consensus sequence, and a poly-A tail following a full SINE-R region on the 3’ end (Fig. 2). SVAs initiating or terminating at an alternate point compared to the subfamily consensus sequence were classified as 5’ and/or 3’ truncation events, and the region of truncation (e.g., SINE-R) was recorded.

To investigate subfamily differences, the hexameric (SVA_D-F), VNTR (SVA_D-F_1_), and poly-A tail (SVA_D-F_1_) regions were analyzed. To capture the broad pattern variability in the hexameric region, all observed patterns were included, given at least one repeat unit was present. For the VNTR analysis, only insertions with a full-length (i.e., not truncated within the region) VNTR were included. To determine poly-A tail lengths, the terminal polyadenylation signal was identified, and the downstream poly-A tail was quantified through the last nucleotide preceding the 3’ TSD.

### Identification and analysis of transductions

All non-reference SVA insertions, plus 500 bp of up- and downstream flanking sequence, were aligned and screened for the presence of sequence 5’ and/or 3’ of the consensus, enclosed within TSDs. Each sequence was manually scrutinized to facilitate identification of the shortest TDs. Putative TD sequences were used as a BLAT [37] query against GRCh38 to identify the origin locus. BLAT hits were filtered by percent identity, and absence of a poly-A tail following the sequence match at the putative origin site was confirmed. GENCODE (v46lift37) [39] and RepeatMasker (v3.0.1) [38] annotations of GRCh38 were used to determine if TD sequences were genic and/or contained TE sequences. Sequence from each putative origin site was retrieved from GRCh38 and aligned with the corresponding non-reference TD sequence to further validate the source locus and determine the exact length of the matching sequence. In some cases, one SVA carried multiple TD sequences traced to different source loci, deemed “concatenated TDs.” These were counted as one event for the overall TD rate, although each separate TD within was recorded. Two or more SVAs with shared TD sequence were sorted into groups. A Chi-square Goodness-of-Fit test was performed to determine if TDs originate in genes more frequently than expected, based on a total genomic proportion of 50.2% genic (protein- and non-protein-coding) sequence [48].

### Source elements

To identify retrotranspositionally active SVA insertions (i.e., source elements), the RepeatMasker track (v3.0.1) [38] of the UCSC Genome Browser (GRCh38) was used to determine presence of a reference SVA element up-or downstream of each TD origin site. The T2T (CHM13v2.0/hs1) assembly was then queried to locate additional source SVAs absent from GRCh38. Finally, TD origin coordinates were screened against all non-reference SVA insertion coordinates to identify non-reference elements serving as source elements. All candidate source elements were confirmed via identification of source TSDs within the TD sequence. A chromosome ideogram was generated based on source element insertion coordinates (GRCh38), using the PhenoGram graphical interface (https://visualization.ritchielab.org/phenograms/plot) [72].

Next, coordinates of the identified source elements were screened against the Freeze 4 DEL callset, which contains loci absent in at least one individual relative to GRCh38 (i.e. polymorphic source elements). For all elements located in the Freeze 4 DEL callset, sequences were aligned to the corresponding source SVA sequence downloaded from the UCSC Genome Browser (GRCh38) for validation, and were classified as polymorphic. Identified source elements absent from the Freeze 4 DEL callset were classified as fixed.

To determine if source elements were enriched within protein-coding genic regions (i.e., insertions in introns), a Chi-square Goodness-of-Fit test was performed. The proportion of the genome composed of protein-coding genes (0.41, including 40% intronic sequence and 1% exonic sequence) [40] was multiplied by the number of non-reference SVAs (543) to calculate expected counts.

### Allele frequencies

Offspring from three parent–child trios (HG00514, HG00733, NA19240) were excluded, and complete genotype information was available for 540/543 SVAs. Allele frequencies were calculated by adding all alleles present for any given insertion and dividing by total number of alleles (*n* = 64).

## Supplementary Information

Supplementary Material 1.

Supplementary Material 2.

## Data Availability

Data generated by the HGSVC2 is publicly available on the online portal: https:/www.internationalgenome.org/data-portal/data-collection/hgsvc2 The dataset generated and analyzed during this study is included within the supplementary material.

## Abbreviations

BLAT: BLAST-like alignment tool
bp: Base pairs
HGSVC: Human Genome Structural Variation Consortium
indel: Insertion/deletion
kb: Kilobase
L1ME-AID: L1 Mediated Annotation and Insertion Detector
LINE: Long interspersed element
MSA: Multiple sequence alignment
MUSCLE: Multiple sequence comparison by log-expectation
NMD: Nonsense-mediated decay
SINE: Small interspersed element
SINE-R: SVA region derived from the 3’ end of the *env* gene and 3’ LTR of HERVK-10
SNV: Single nucleotide variant
SVA: SINE variable number tandem repeat *Alu*
TD: Transduction
TE: Transposable element
TSD: Target site duplication
UTR: Untranslated region
VNTR: Variable number tandem repeat
