# Supplementary material for: Structural and transduction patterns of human-specific polymorphic SVA insertions
Source: Mob DNA. 2025 Nov 6;16:42. doi: 10.1186/s13100-025-00373-w (PMC12593883; doi:10.1186/s13100-025-00373-w)
Supplement: Supplementary file 2 — Supplementary Material 2. [file 13100_2025_373_MOESM2_ESM.docx]

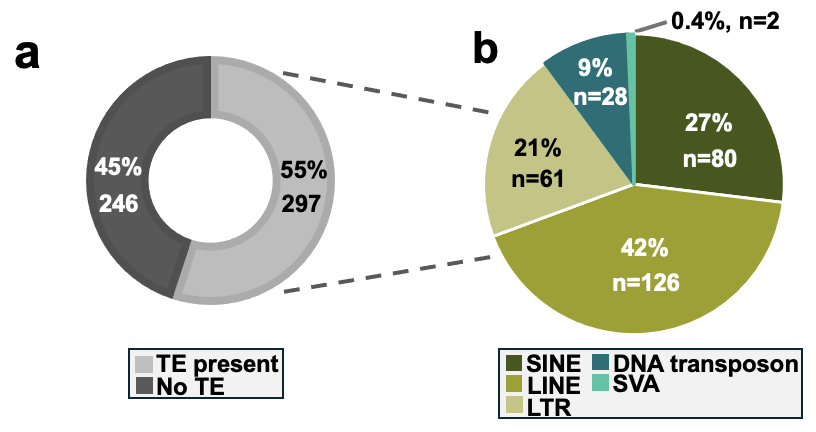


**Figure S1. TE sequence at SVA insertion sites.** a) TE presence/absence at SVA insertion sites. We scrutinized the insertion site of all 543 non-reference SVA elements and found that 55% (297/543) inserted within TE sequence, while 45% (246/543) did not. b) Investigating further, we determined that, overall, 23% inserted into LINEs (126/543), 15% (80/543) into SINEs, 11% (61/543) into LTRs, 5% (28/543) into DNA transposons, and 0.4% (2/543) into other SVAs**.** This largely corresponds to background TE composition in the human genome, with slight enrichment across all TE subfamilies.

**
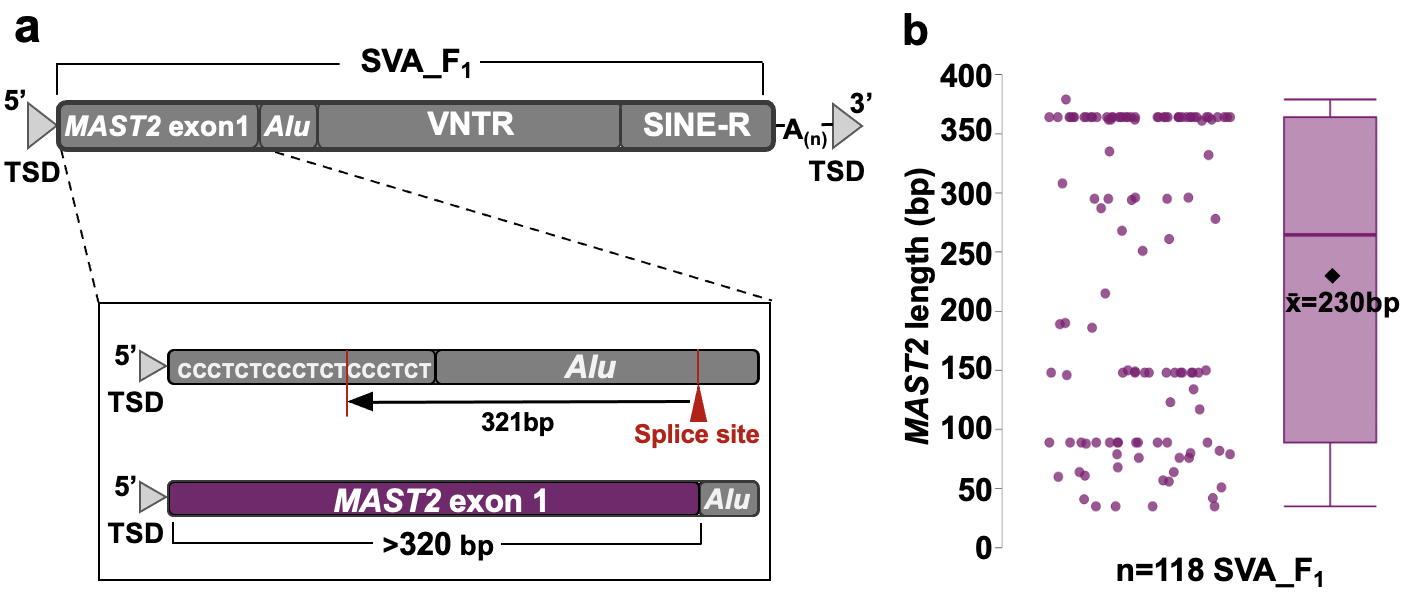
**

**Figure S2. Analysis of SVA_F_1_ *MAST2* sequence.** a) Full-length vs. truncated SVA_F_1_. We defined full-length for SVA_F_1_ based on the distance (321bp) from the *MAST2*/SVA splice junction (AG/CC) to the first nucleotide of the first hexameric repeat, relative to the SVA_F consensus sequence. Thus, full length was described as >320bp of *MAST2* sequence on the 5’ end, in conjunction with a poly-A tail following a full SINE-R region on the 3’ end. This is based on previous literature defining full-length for SVA subfamilies A-F as the presence of at least one hexameric unit on the 5’ end, as well as a poly-A tail following the SINE-R region on the 3’ end. We considered establishing full-length as 364bp, relative to the length of *MAST2* sequence observed in chromosome 10-derived elements that are insulated on their 5’ ends by an *Alu* TD. However, we noted one insertion surpassing this length, with 379bp of *MAST2* sequence, and lengths up to 383bp have been documented in other studies (Bantysh and Buzdin, 2009). b) Length of *MAST2* sequences within SVA_F_1_ elements. Each data point represents a unique SVA_F_1_ insertion. *MAST2* lengths ranged from 35bp-379bp, with an average of 230bp. Clustering around 364bp represents chromosome 10-derived elements with a 5’ *Alu* insulating the *MAST2* sequence.


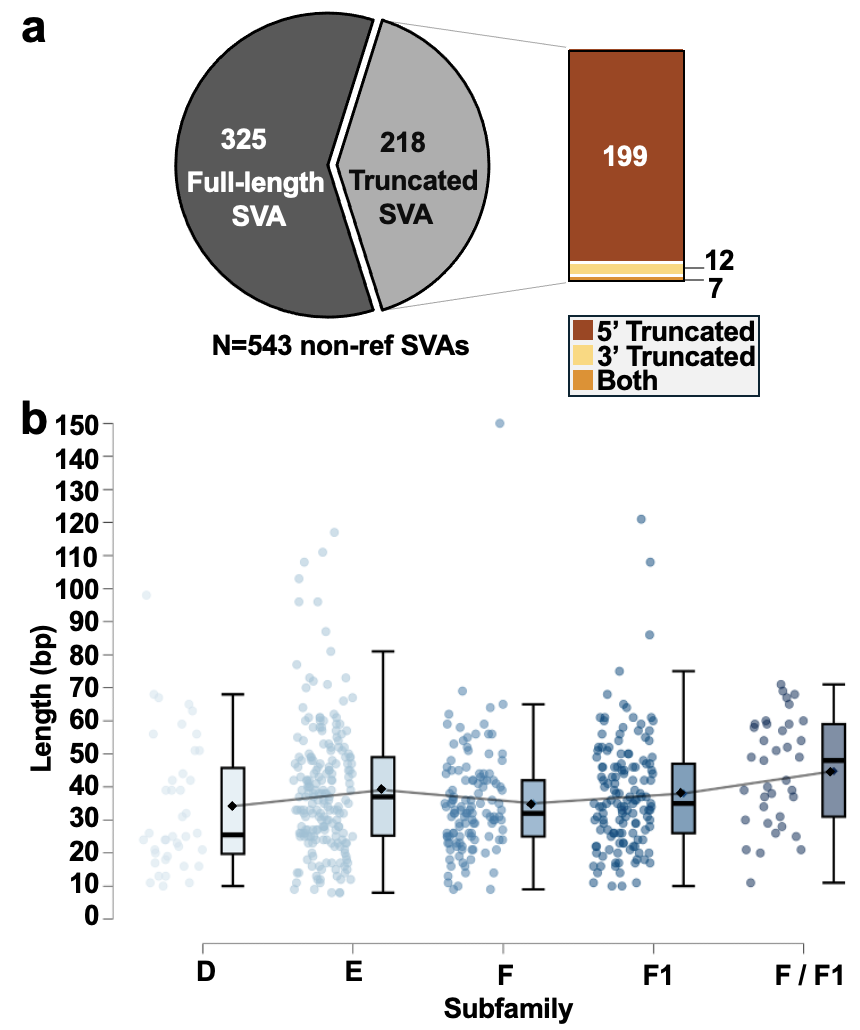


**Figure S3. SVA truncation and polyA-tail analysis.** a) We observed 325 (59.9%) full-length SVA elements and 218 (40.1%) truncated SVA elements. As expected, the vast majority (91%) of truncations were observed on the 5’ end within the *Alu*, VNTR, and SINE-R regions. Of the 19 observed 3’ truncations, 18 occurred within the SINE-R region and one occurred within the VNTR. b) Variation in poly-A tail length across subfamilies.

**Figure S4. Occurrence of SVA-mediated TD events.** a) Proportion of polymorphic SVA elements in each subfamily that harbor a TD. Excluding SVA_F_1_, TD rate appears to be directly correlated with evolutionary age of the subfamily. b) Comparison of poly-A tail lengths among non-reference SVAs without TD, with a 5’ TD, and with a 3’ TD. Bars denote statistically significant relationships (**p<0.001).


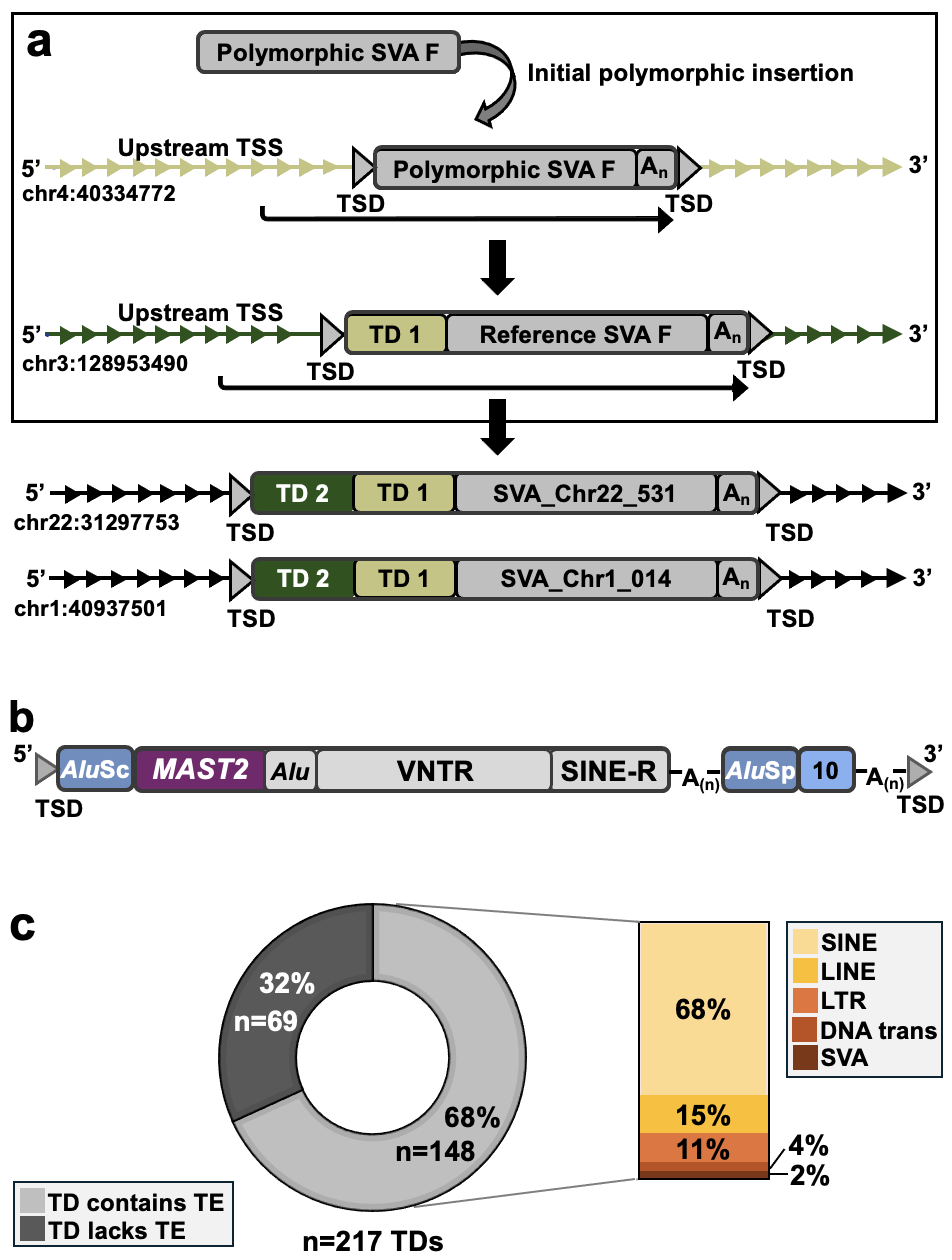


**Figure S5. SVA-mediated TD events.** a) Example of an SVA carrying a concatenated TD, with two distinct sequences traced to loci on chromosomes 4 and 3. Initial insertion of a polymorphic SVA_F likely occurred upstream of the TD origin on chromosome 4. There is no fixed insertion at this locus in GRCh38. This SVA acquired the initial 5’ TD (light green) and generated a copy which retrotransposed to chromosome 3. This copy then acquired an additional 5’ TD (dark green) and produced at least two offspring (SVA_Chr22_531 and SVA_Chr1_014) on chromosomes 22 and 1, present in our non-reference dataset. b) Structure of an SVA_F_1_ derived directly or indirectly from chromosome 10. These elements are characterized by inclusion of *Alu* TD sequence on their 5’ and/or 3’ ends (Hancks et al, 2009) (Damert et al., 2009), and often include additional chromosome 10 sequence downstream of the 3’ *Alu*. We identified 50 chromosome 10-derived elements with a 3’ *Alu* transduction, of which 96% (48) contained additional 3’ sequence, ranging from 80bp to 163bp (M: 92bp, MDN: 81bp). Of the 87 total SVA_F_1_ TDs, 65 include sequence derived from chromosome 10 (5’=15, 3’=28, both=22). Thus, this group comprises the majority (75%; 65/87) of SVA_F_1_ TD events. c) TE sequences identified within TDs. We find that 68% (148/217) of TDs contain TE sequence. Of these, 25 harbor more than one TE sequence, resulting in a total of 215 distinct TE sequences included within 148 separate TD events. The largest proportion of transduced TE sequences are SINE (67.9%; 146/215), followed by LINE (14.9%; 32/215), LTR (11.2%; 24/215), DNA transposon (3.7%; 8/215), and SVA (2.3%; 5/215). Compared to TE proportions in the genome, we find an enrichment (~5x) for inclusion of *Alu* sequence within TDs.

**
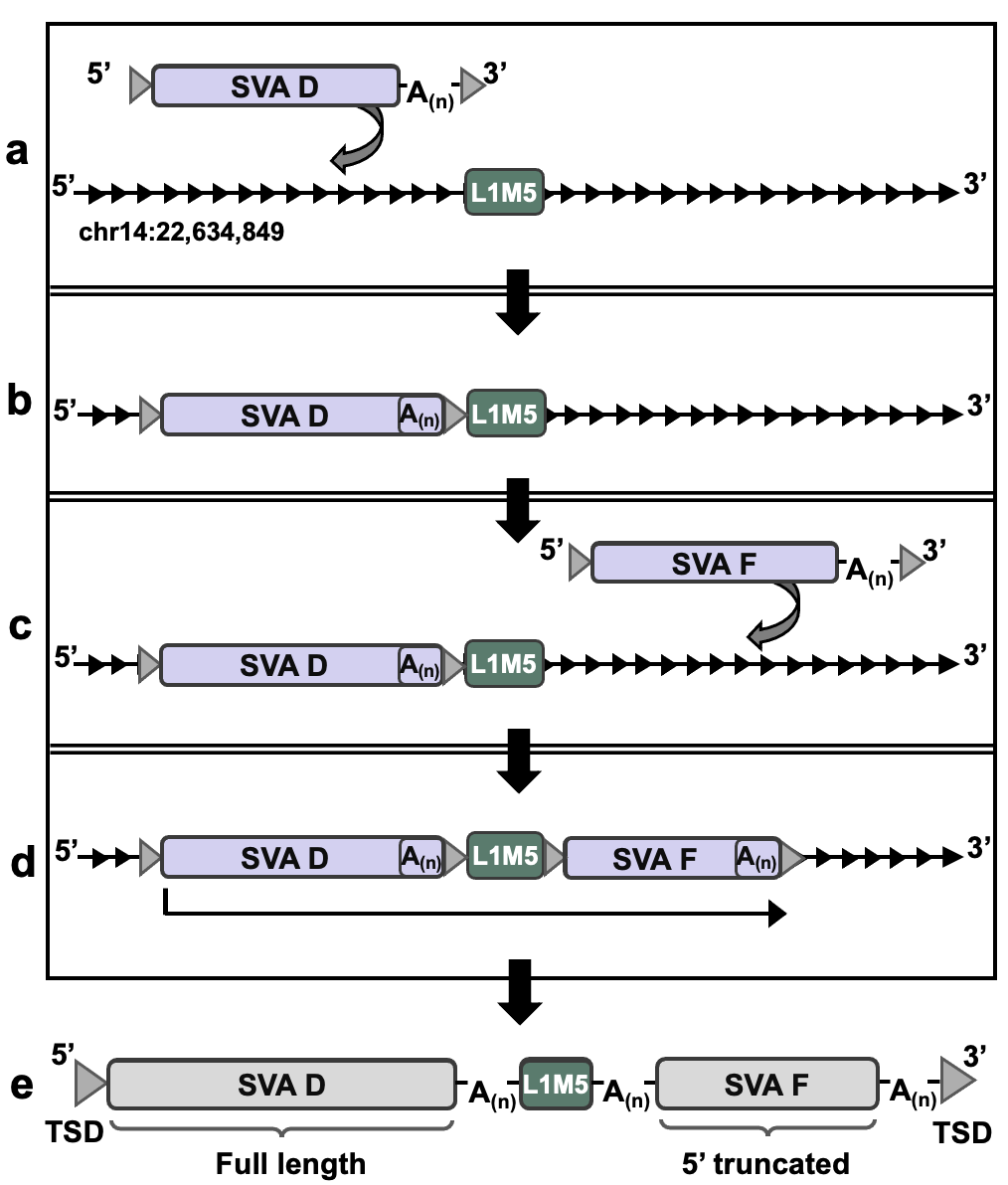
**

**Figure S6. Generation mechanism and structure of the concatenated SVA element group.** A BLAT (Kent, 2002) query of the 3’ TD sequence revealed a putative source locus on chromosome 14 (chr14:22,634,849), where reference SVA_D and SVA_F elements reside on either side of an L1M5. **a-c)** We determined that the SVA_D and SVA_F elements inserted independently (confirmed by the presence of unique TSDs enclosing each). Searching panTro6, we identified the L1M5 at its orthologous site, but found that both SVAs are absent, indicating that this is a human-specific event. **d)** The SVAs and L1M5 were transcribed together, substantiated by unique TSDs enclosing all three elements as a unit. Based on our finding that 3’ TD events occur twice as often as 5’ events, the propensity of 3’ TDs to be longer than 5’ TDs, and the fact that the SVA_D is full-length suggest that the L1M5 and SVA_F sequences are carried by the SVA_D as a 3’ TD. **e)** We identified six non-reference SVA insertions in our dataset exhibiting the concatenated chromosome 14 structure.


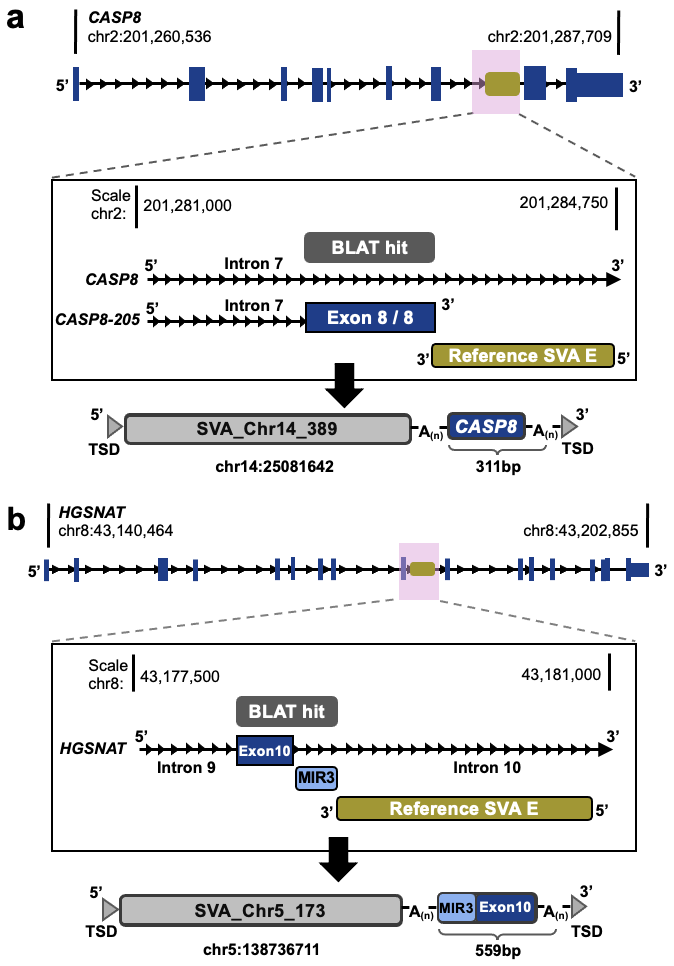


**Figure S7. Previously reported 3’ exonic TD events of *CASP8* and *HGSNAT*.** a) *CASP8*, located on chromosome 2, encompasses 9 exons and spans 52.3kb. A BLAT (Kent, 2002) search revealed that two SVAs in our dataset carry TDs comprised of *CASP8* sequence. At the TD origin locus, we identified a reference SVA_E insertion oppositely oriented to the gene. Bypassing of the reference SVA’s internal polyadenylation signal and readthrough into *CASP8* sequence likely occurred. b) The *HGSNAT* gene is located on chromosome 8 and contains 18 exons spanning a total of 62.4kb. The mechanism most likely attributed to the acquisition of *HGSNAT* exonic sequence is as described for *CASP8*.


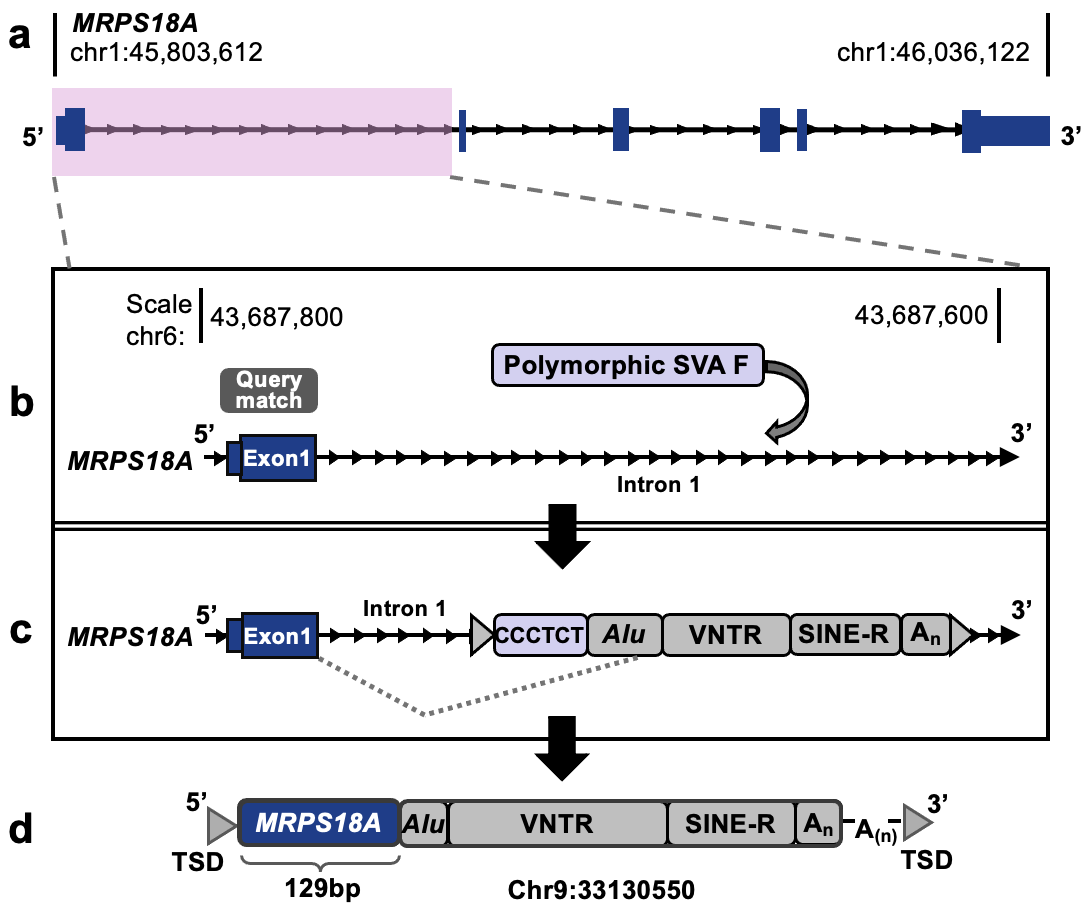


**Figure S8. Generation of *MRPS18A-SVA*.** a) The protein-coding *MRPS18A* gene, located on chromosome 6, contains 6 exons and spans a total length of 16.6kb. b) Based on BLAT analysis (Kent, 2002), we identified the TD sequence as 5bp upstream of the *MRPS18A* start site and the entirety of exon 1. There is no reference SVA at this locus in GRCh38. Given this, we propose that a polymorphic SVA_F inserted into intron 1 in the same orientation as the gene, introducing a cryptic splice site. c) Alternative splicing occurred between the 3’ end of exon 1 and the same splice site within the *Alu* region used in the generation of SVA_F_1_. d) The chimeric *MRPS18A*-SVA (SVA_Chr9_273) in our dataset was generated and inserted on chromosome 9. A BLAT query of the T2T assembly did not reveal any additional *MRPS18A*-SVA copies.


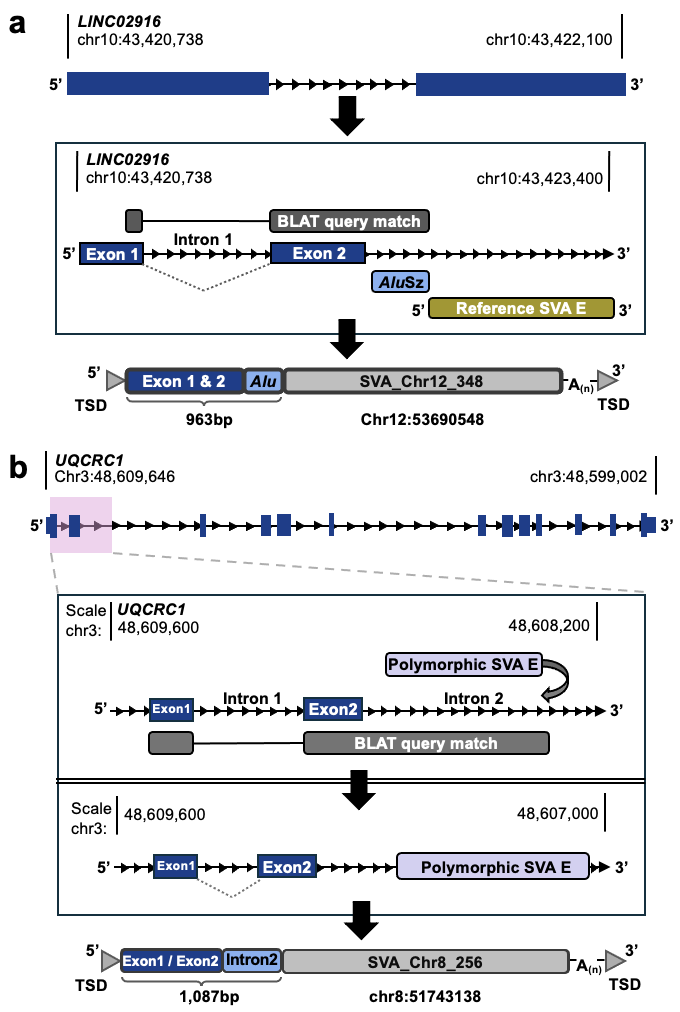


**Figure S9. Examples of transduction events generated by read-through.** a) *LINC02916*, a lincRNA gene located on chromosome 10, is composed of two exons and spans 1.4kb. TD sequence 5’ of SVA_Chr12_348 was found to match most closely to *LINC02916* exons 1 and 2, as well as an *Alu*S within intron 2*.* There is a reference SVA_E located just downstream from the origin locus in GRCh38, in the same orientation as the gene. Transcription likely initiated at the *LINC02916* TSS. Splicing appears to have occurred at canonical splice sites between exon 1 and 2, followed by readthrough into intron 2 and the reference SVA_E. The observed non-reference insertion, SVA_Chr12_348, was generated and inserted on chromosome 12, carrying a 963bp 5’ transduction from *LINC02916.* b) 5’ TD of *UQCRC1* sequence. *UQCRC1* is a protein-coding gene located on chromosome 3 that contains 13 exons and spans a total of 10.6kb. A BLAT search (Kent, 2002) revealed that the TD sequence carried by SVA_Chr8_256 matched exon 1, exon 2, and intron 2 of *UQCRC1*. Although there is no reference insertion at this locus, we speculate that a polymorphic SVA_E inserted into intron 2, in the same orientation as the gene and was transcribed with *UQCRC1*. Splicing occurred at canonical splice sites between exons 1 and 2, and readthrough occurred into intron 2 and the polymorphic SVA_E. SVA_Chr8_256 was generated and inserted on chromosome 8, bringing with it a 1,087bp 5’ TD from *UQCRC1*.


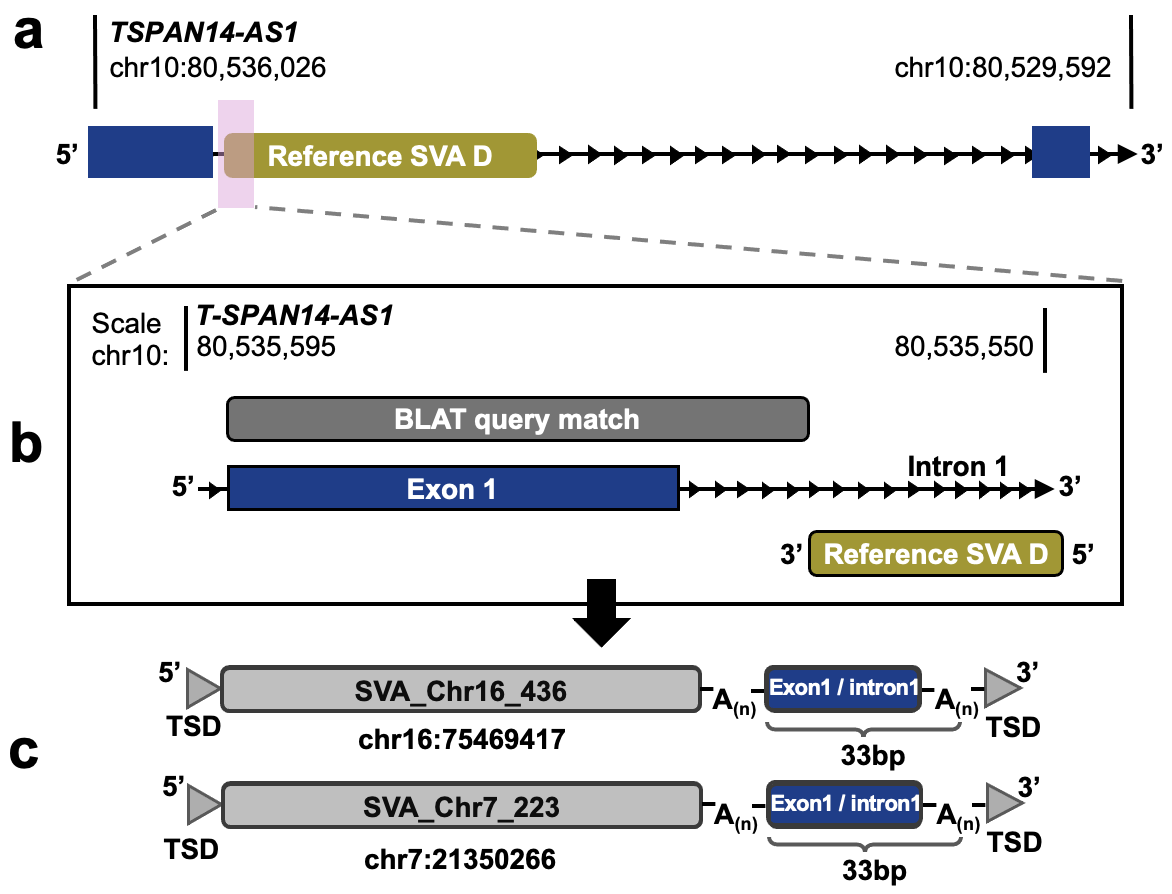


**Figure S10.** ***TSPAN14-AS1* exonic 3’ TD event.** *T-SPAN14-AS1* is an antisense long non-coding RNA gene located on chromosome 10 that contains two exons and spans 6.4kb in length. A BLAT search (Kent, 2002) traced the TD origin locus to intron 1 and exon 1 of *T-SPAN14-AS1*. A reference SVA_D, oppositely oriented to the gene, is present in intron 1 of *T-SPAN14-AS1* (GRCh38). It is likely that transcription initiates at the 5’ end of the SVA, bypasses its internal termination signal, and continues through intron 1 and into exon 1. In addition to SVA_Chr16_436 and SVA_Chr7_223, which both contain exon 1 sequence, there are six additional non-reference insertions in our dataset that contain TDs of only the intronic sequence. We have also located two reference insertions on chromosomes 9 and 20 (chr9:88243898 and chr20:36999350; GRCh38) that both harbor the intronic transduction. We can infer that one of our non-reference insertions (SVA_Chr7_222) comes from the reference insertion on chromosome 20, as it harbors a 75bp 5’ TD from this locus, in addition to the 3’ TD of *T-SPAN14-AS1* intron 1 sequence. Thus, the chromosome 20 element likely represents a second source locus for this group, contributing to its expansion.


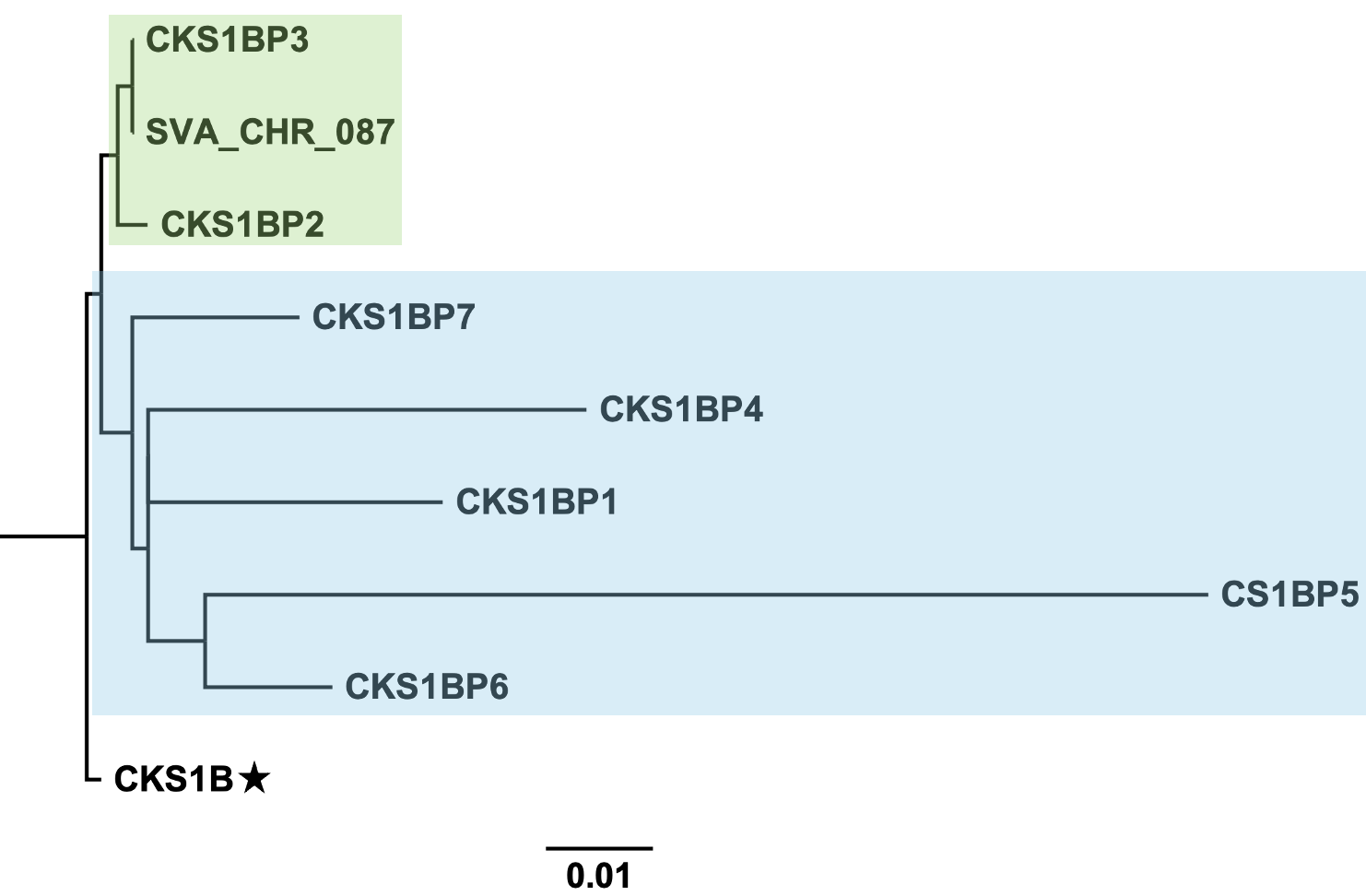


**Figure S11. SVA-*CKS1BP* maximum likelihood tree.** Phylogenetic analysis of all *CKS1BP* copies and the *CKS1B* mRNA reveals evolutionary relationships. The maximum likelihood tree, generated from a MSA of only the sequences shared between the *CKS1BP* copies and the *CKS1B* mRNA, indicates that SVA-associated and non-SVA-associated *CKS1BP* copies form two distinct clades. This, in conjunction with a BLAT query to investigate presence of *CKS1BP* at orthologous loci in PanTro6, supports two distinct origin events, with non-SVA-associated copies being of more ancient origin.
